# The RNA-binding protein LRPPRC promotes resistance to CDK4/6 inhibition in lung cancer

**DOI:** 10.1038/s41467-023-39854-y

**Published:** 2023-07-14

**Authors:** Wei Zhou, Wenxi Wang, Yuxin Liang, Ruibin Jiang, Fensheng Qiu, Xiying Shao, Yang Liu, Le Fang, Maowei Ni, Chenhuan Yu, Yue Zhao, Weijia Huang, Jiong Li, Michael J. Donovan, Lina Wang, Juan Ni, Dachi Wang, Ting Fu, Jianguo Feng, Xiaojia Wang, Weihong Tan, Xiaohong Fang

**Affiliations:** 1 Hangzhou Institute of Medicine (HIM), University of Chinese Academy of Sciences (Zhejiang Cancer Hospital), Chinese Academy of Sciences, Hangzhou, Zhejiang 310022 PR China; 2 Beijing National Research Center for Molecular Sciences, Institute of Chemistry, Key Laboratory of Molecular Nanostructure and Nanotechnology, Chinese Academy of Science, Beijing, 100190 PR China; 3 School of Molecular Medicine, Hangzhou Institute for Advanced Study, UCAS, Hangzhou, 310024 PR China; 4 University of Chinese Academy of Sciences, Beijing, 100049 PR China; 5 Department of Medicinal Chemistry, Massey Cancer Center, Philips Institute for Oral Health Research, Virginia Commonwealth University, Richmond, VA 23298-0540 USA

**Keywords:** Translational research, Cancer therapeutic resistance

## Abstract

Kinase inhibitors against Cyclin Dependent Kinase 4 and 6 (CDK4/6i) are promising cancer therapeutic drugs. However, their effects are limited by primary or acquired resistance in virtually all tumor types. Here, we demonstrate that Leucine Rich Pentatricopeptide Repeat Containing (LRPPRC) controls CDK4/6i response in lung cancer by forming a feedback loop with CDK6. LRPPRC binds to *CDK6*-mRNA, increasing the stability and expression of CDK6. CDK6 and its downstream E2F Transcription Factor 1 (E2F1), bind to the *LRPPRC* promoter and elevate *LRPPRC* transcription. The activation of the LRPPRC-CDK6 loop facilitates cell cycle G1/S transition, oxidative phosphorylation, and cancer stem cell generation. Gossypol acetate (GAA), a gynecological medicine that has been repurposed as a degrader of LRPPRC, enhances the CDK4/6i sensitivity in vitro and in vivo. Our study reveals a mechanism responsible for CDK4/6i resistance and provides an enlightening approach to investigating the combinations of CDK4/6 and LRPPRC inhibitors in cancer therapy.

## Abstract (web summary)

CDK4/6 inhibition is a promising therapeutic approach to treat cancer, but it is challenged by resistance development. Here, the authors show that the RNA binding protein LRPPRC forms a positive feedback loop with CDK6 and inhibiting LRPPRC with the FDA-approved gossypol acetate overcomes CDK4/6 inhibition resistance.

## Introduction

Uncontrolled cell division and proliferation are fundamental hallmarks of cancer^1^. Targeting cyclin-dependent kinase 4 and 6 (CDK4/6), the proteins controlling cell cycle entry, has become an attractive tumor treatment strategy for RB Transcriptional Corepressor 1 (RB1) wild-type patients^2,3^. However, completed clinical trials have shown that monotherapy using CDK4/6 inhibitors (CDK4/6i) is not effective due to primary resistance or rapidly acquired resistance^4,5^. Developing CDK4/6i combination strategies to overcome this resistance is crucial for successful treatment. Recently, three CDK4/6i (palbociclib, abemaciclib, and ribociclib) have received FDA approval to treat estrogen receptor (ER)-positive, Erb-B2 Receptor Tyrosine Kinase 2 (HER2)-negative advanced breast cancer in combination with endocrine therapy^6,7^. In this context, ER signal could enhance CDK4/6 kinase activity by increasing the expression of cyclin D, and overexpressed cyclin D promotes the resistance to endocrine therapy. This enables the synergistic effect of ER inhibitors and CDK4/6i^8,9^. While most tumors, such as lung cancer, are hormone-independent, and this combination strategy is not generalizable. As far as we know, hundreds of clinical trials have been conducted to test various potential combinations with CDK4/6i, including chemotherapy, targeted therapy, and immunotherapy^10–12^. Unfortunately, none of these attempts have achieved similar effects observed in ER-positive breast cancer.

The aberrant overexpression of CDK6 protein is the most recognized reason for CDK4/6i primary and acquired resistance for many cancers, including lung cancer^13–15^. In clinical practice, lung cancer patients expressing a high level of CDK6 showed significant resistance towards CDK4/6i^16^. Overexpressed CDK6 protein could facilitate cells to activate E2F Transcription Factor 1 (E2F1) to directly initiate the cell cycle G1/S transition. In addition, the latest researches showed that CDK6, but not CDK4, participates in several cellular processes in a kinase-independent manner, including cell cycle G1/S transition, which further promoting drug resistance and cell survival^17,18^. Although the importance of CDK6 in CDK4/6i resistance is widely recognized, the mechanism of CDK6 overexpression remains largely unclear, especially in tumors other than breast cancer. There is still no effective strategy to overcome the CDK6-mediated CDK4/6i resistance^19^. Even the latest CDK4/6 degraders developed by the Proteolysis-Targeting Chimeras strategy are ineffective in binding to CDK6 in resistant cells, because CDK6 is expressed in a thermostable state that interacted with the HSP90–CDC37 complex^16,20^. Therefore, further exploration of the mechanisms underlying CDK6 overexpression and the development of combination strategies to downregulate CDK6 expression have significant clinical value for overcoming CDK4/6i resistance in hormone-independent cancers, such as lung cancer.

Herein, we report that Leucine-rich pentatricopeptide repeat-containing (LRPPRC) protein can form a positive feedback loop with CDK6 to regulate CDK6 expression and CDK4/6i treatment response in lung adenocarcinoma (LUAD). LRPPRC is an unclassical RNA-binding protein that has been recently found overexpressed in many tumors^21–24^. Reported molecular functions of LRPPRC include cell oxidative phosphorylation (OXPHOS) regulation and cancer stem cells (CSCs) maintenance^24–26^. This work reveals a function of both LRPPRC and CDK6 in tumor development and CDK4/6i resistance. Mechanistically, cytoplasmic LRPPRC selectively binds to *CDK6* mRNA, increases *CDK6* stability, and promotes CDK6 protein expression. Meanwhile, both CDK6 and E2F1 can bind to the promoter of *LRPPRC* and increase the expression of LRPPRC, thus further promoting cellular OXPHOS and CSCs generation to aggravate CDK4/6i resistance. Gossypol acetate (GAA), a clinically-approved gynecological old drug and recently-reported LRPPRC-specific degrader^27,28^, has been demonstrated to inhibit LRPPRC-CDK6 positive loop in this work. We use the combination of GAA and CDK4/6i to down-regulate CDK6 and LRPPRC simultaneously, thereby inhibiting cell cycle G1/S transition, suppressing OXPHOS subunit synthesis, and eliminating CSCs. This combination has shown significant tumor-suppressive effects with both cell line-derived xenograft and patient-derived xenograft (PDX) models. We have discovered a positive loop in CDK4/6i resistance and provided a promising intervention to address CDK4/6i resistance in LUAD.

## Results

### GAA restrained CDK6 expression and increased CDK4/6i sensitivity in vitro

**Fig. 1:**
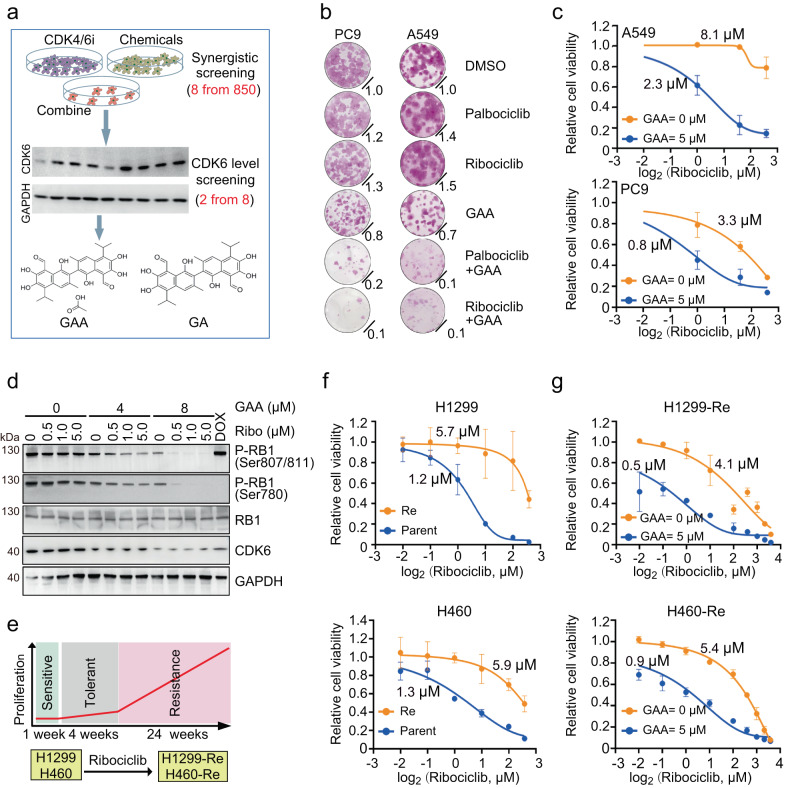
GAA decreased CDK6 expression and increased CDK4/6i sensitivity in vitro. **a** Schematic diagram of the screening process to search for chemical compounds which inhibited the expression of CDK6 protein and increased the efficacy of CDK4/6i. Eight compounds were screened out from 850 natural product compounds, with a coefficient of drug interaction score below 0.8 when combined with CDK4/6i ribociclib. Then, two of the eight compounds could reduce CDK6 expression in immunoblotting by about 50%. **b** Representative colony formation images of LUAD cell lines treated with indicated compounds. Normalized crystal violet staining intensity (mean; *n* = 3 biological replicates) was displayed in the lower right corner. **c** Dose-response curves of A549 and PC9 cells to CDK4/6i with or without GAA treatment (mean ± SEM.; *n* = 4 biological replicates). **d** Immunoblotting of indicated proteins in LUAD cells. LUAD cells were pretreated with ribociclib (Ribo), doxorubicin (DOX), and gossypol acetate (GAA) at different concentrations for 48 hours. **e** Schematic of generating LUAD cell lines with acquired resistance against CDK4/6i through continuous cell culture and stepwise dose escalation of ribociclib from 500 nM to 20 μM. Cell lines are listed below. Cells were first co-cultured with 500 nM ribociclib for one week, then co-cultured with 1 μM ribociclib for four weeks, 5 μM, 10 μM, 15 μM, and 20 μM ribociclib for six weeks sequentially and respectively. **f** Dose-response curves of parent cells (Parent) and acquired resistance cells (Re) to ribociclib (mean ± SEM*, n* = 4 biological replicates). **g** Dose-response curves of acquired resistance cells to ribociclib with or without GAA treatment (mean ± SEM, *n* = 3 biological replicates in H1299 cell lines and *n* = 4 biological replicates in H460 cell lines). Images **b** and **d** are representative results of *n* = 3 independent experiments with similar tendency. Source data are provided as a Source Data file.

We designed a two-step screening system to identify the chemicals which could downregulate CDK6 protein and synergize with CDK4/6i to inhibit the growth of LUAD cells (Fig. 1a). Two LUAD cell lines, A549 and PC9, were used since they showed a high CDK6:CDK4 ratio, harbored a robust RB1 function and were more resistant against CDK4/6i compared to other LUAD cells (Supplementary Fig. S1a-c)^16^. In the first synergistic effect screening, eight compounds were retained from 850 bioactive natural products, with a coefficient of drug interaction score (CDI) below 0.8 when combined with CDK4/6i ribociclib (Supplementary Fig. S1d-f). After the second immunoblotting screening, only two of the eight compounds reduced CDK6 protein by more than 50% in A549 cells: gossypol and its medicinal form, gossypol acetate (GAA, Supplementary Fig. S2a-d). The colony-forming assays showed that the combination of GAA with either palbociclib or ribociclib could reduce the tumor colony by about 90%, and the suppression effect was much higher than single-agent treatment (Fig.1b). CCK8 cell viability assay showed that 5 μM GAA lowered the IC50 value against ribociclib and palbociclib (Fig. 1c and Supplementary Fig. S2e, f) in both A549 and PC9 cells. At the molecular level, either GAA or CDK4/6i alone could only weakly reduce the phosphorylation of RB1 protein (Ser807/811 and Ser780), the direct target of CDK4/6. Nevertheless, the combination of the two compounds resulted in the depletion of phosphorylated RB1 to an undetectable level (Fig. 1d).

We also constructed acquired resistance models against CDK4/6i ribociclib in vitro by stepwise dose escalation of CDK4/6i using two CDK4/6i sensitive LUAD cell lines, H1299 and H460 (Fig. 1e, Re denotes ribociclib resistant cell lines, H1299-Re and H460-Re). There was about five-fold increase in IC50 against both ribociclib and palbociclib for each acquired-resistant cell line (Fig. 1f and Supplementary Fig. S3a, b). Acquired-resistant cells expressed a higher level of CDK6, phosphorylated RB1, and could proliferate well at a high concentration of ribociclib (10 μM, Supplementary Fig. S3c-f). The addition of GAA also down-regulated CDK6 protein expression and reduced the phosphorylated RB1 synergistically in combination with ribociclib (Supplementary Fig. S3g). The CCK8 assay suggested that GAA decreased the IC50 values of CDK4/6i in the acquired-resistant cells to a similar value of the parental cells (Fig. 1g and Supplementary Fig. S3h), which was further supported by cell proliferation assay and colony-forming assays (Supplementary Fig. S3i, j). Therefore, we concluded that GAA suppressed CDK6 expression and restored sensitivity against CDK4/6i in acquired-resistant models.

### GAA regulated CDK4/6i sensitivity by targeting LRPPRC

Several protein targets have been reported as potential intracellular targets of GAA, mainly focused on anti-apoptotic proteins (B-Cell CLL/Lymphoma 2 (Bcl-2), Bcl-2-Like Protein 1 (Bcl-xL), Myeloid Cell Leukemia Sequence 1 (Mcl-1), and Apurinic/Apyrimidinic Endodeoxyribonuclease 1 (APEX1))^29–31^. However, at the concentration of GAA showing an apparent synergistic effect with CDK4/6i, we failed to detect cell apoptosis by either Annexin V staining or immunoblotting of apoptosis protein markers (Supplementary Fig. S4a-c). In addition, ABT-737 (a well-known BCL2-specific inhibitor) did not show any synergistic effect in combination with CDK4/6i (Supplementary Fig. S4d, e), and the protein level of CDK6 remained unchanged after ABT-737 treatment (Supplementary Fig. S4f). These results indicated GAA modulated CDK4/6i sensitivity in an apoptotic-independent manner.

**Fig. 2:**
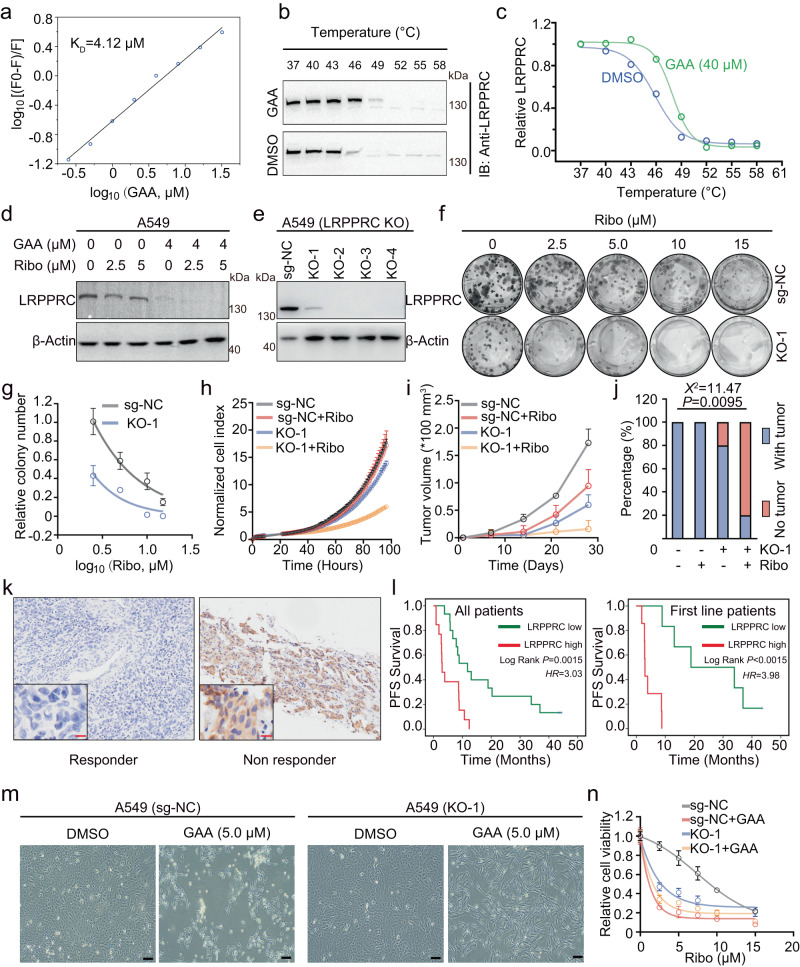
GAA regulated CDK6 expression and CDK4/6i sensitivity via LRPPRC. **a** Fluorescence titration analysis of the binding between LRPPRC protein and GAA, where *F*_*o*_ is the fluorescence intensity of protein, *F* is the fluorescence intensity of protein after the addition of ligand. **b, c** Cellular thermal shift assay analysis of LRPPRC in A549 cells treated with DMSO or 40 μM GAA. **d** Immunoblotting of LRPPRC protein in A549 cells treated with ribociclib and GAA. **e** Immunoblotting of LRPPRC protein in A549 cells before and after LRPPRC knockout. **f, g** Representative images and quantified cell colonies of A549 cells (before and after LRPPRC knockout) treated with different concentrations of ribociclib (mean ± SEM.; *n* = 3 biological replicates). **h** Cell proliferation curves of A549 (before and after LRPPRC knockout) in the presence of ribociclib (Ribo, 5 μM; mean ± SD.; *n* = 3 biological replicates). **i, j** Tumor proliferation curves of A549 cells (before and after LRPPRC knockout) treated with ribociclib (mean ± SEM.; *n* = 5 mice in each group). The tumor formation rate in different groups was calculated. **k** IHC staining of LRPPRC in breast cancer patients responding or not responding to palbociclib treatment. **l** PFS analysis of breast patients who received palbociclib based on LRPPRC expression (*n* = 15 in LRPPRC- low arm, n = 13 in LRPPRC-high arm). PFS of patients who received first-line palbociclib treatment was analyzed separately (*n* = 6 in LRPPRC-low arm, *n* = 7 in LRPPRC- high arm)**. m** Cell morphology imaging of A549 cells (before and after LRPPRC knockout) treated with DMSO or 5 μM GAA for 48 hours. **n** Dose-response curves of A549 cells to ribociclib before and after LRPPRC knockout (mean ± SEM.; *n* = 3 biological replicates). Immunoblotting results in images **b** and **d** were representative results of *n* = 2 and 3 independent experiments, respectively, with similar tendency. IHC images in **k** and cell morphology images in **m** were representative results of *n* = 2 independent experiments with similar tendency. Statistical significance in figure **j** was determined by Chi-square analysis. Statistical significance in figure **l** was determined by Kaplan-Meier analysis. Source data are provided as a Source Data file.

We and other groups have recently found that the LRPPRC protein is a more critical intracellular protein target of GAA in LUAD, where GAA only suppressed the proliferation of LRPPRC-positive LUAD patient-derived tumor xenograft (PDX)^27,28^. LRPPRC is a non-classical RNA binding protein that mainly controls the stability and translation of mRNAs. The fluorescence titration experiments using the purified RNA binding fragment of LRPPRC (C terminals of LRPPRC) confirmed that LRPPRC and GAA interacted directly with a dissociation constant K_D_ of 4.12 μM (Fig. 2a, this value is smaller than that in our previous report since we used the protein with a higher purity this time). The cellular thermal shift assay (CETSA) was carried out to test the interaction of LRPPRC and GAA in cells^32^, which showed that GAA could increase the thermal stabilization of LRPPRC (Fig. 2b, c). Immunoblotting showed a decreased LRPPRC protein level in the cells treated by GAA alone or combined with CDK4/6i (Fig. 2d). These results suggested that GAA could bind to LRPPRC in the complicated cellular environment and induce LRPPRC degradation.

We then constructed LRPPRC stable knockout cell lines with different LRPPRC suppression levels by CRISPR-Cas9 to evaluate the role of LRPPRC in ribociclib resistance (sg-NC is control cells (*LRPPRC* ^+/+^), KO-1 is heterozygote (*LRPPRC*^+/-^), and others are homozygotes (*LRPPRC*^-/-^), Fig. 2e). We selected *LRPPRC*^+/-^ cells to test the CDK4/6i sensitivity in comparison with control cells, both in vitro and in vivo. The in vitro colony-forming assays and cell proliferation experiment indicated that 5 μM ribociclib significantly inhibited *LRPPRC*^+/-^ cells, but showed little effect on the control cells (Fig. 2f-h). In the in vivo subcutaneous tumors model, ribociclib significantly reduced the tumor formation rate and average tumor volume of *LRPPRC*^+/-^ cells, and the anti-tumor effect was much better than that with control cell-generated subcutaneous tumors (Fig. 2i, j). At the clinical level, we verified the roles of LRPPRC in CDK4/6i treatment response by Immunohistochemical (IHC) experiment using samples from breast cancer patients, as breast cancer is the only approved clinical application for CDK4/6i. The IHC results showed that patients with a high level of LRPPRC protein harbored a significantly shorter progression-free-survival time (PFS) during palbociclib therapy, especially for the first-line treated patients (Fig. 2k, l). After treating the cells with 5 μM GAA, we found *LRPPRC*^+/-^ cells remained a healthier morphology than the control cells, and more LRPPRC KO cells survived (Fig. 2m). Furthermore, although GAA significantly reduced the IC50 value of ribociclib with the control cells, it had little effect on *LRPPRC*^+/-^ cells (Fig. 2n). Therefore, LRPPRC played an essential role in CDK4/6i resistance, and GAA regulated CDK4/6i sensitivity mainly by targeting LRPPRC.

### LRPPRC promoted CDK6 expression and accelerated cell cycle G1/S transition

**Fig. 3:**
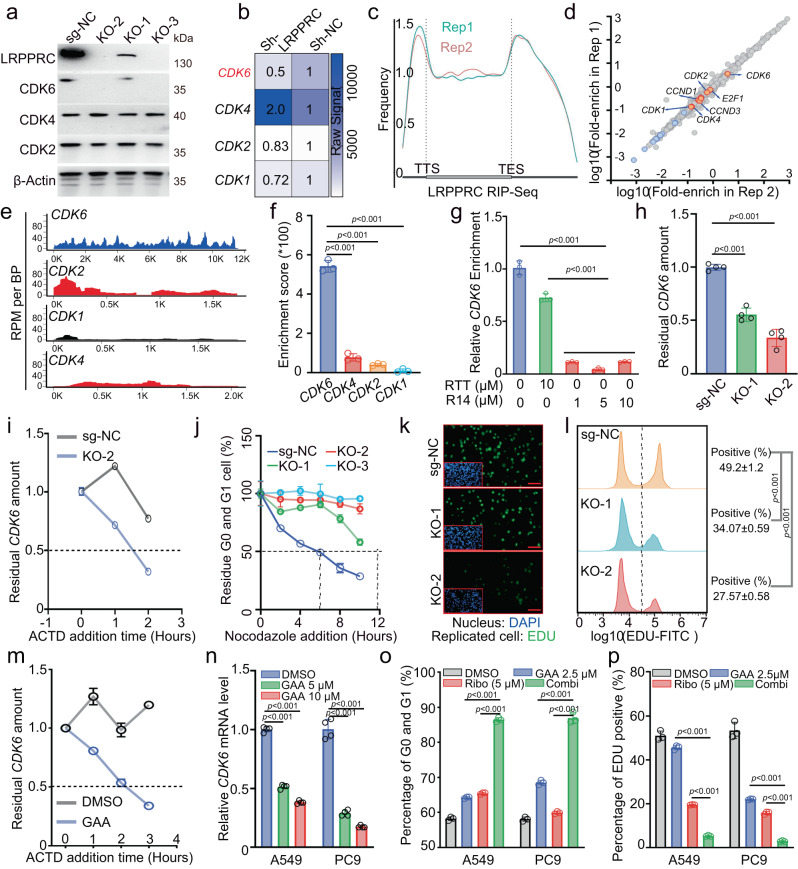
LRPPRC directly modulated CDK6 expression and controlled cell cycle G1/S transition. **a** Immunoblotting of indicated proteins in different A549 cell colonies. **b** Heatmap of relative abundance of indicated transcripts detected by hybridization chip in A549 cells before and after LRPPRC knockdown. **c** Meta-gene analysis showed the distribution of LRPPRC-binding sites along with a normalized transcript. **d** The RIP/Input signal mRNAs determined by two RIP-sequencing biological replicates. **e** Mapping of RIP-seq reads back to the genomic locus of indicated transcripts. **f** RIP/Input signals of indicated mRNAs quantified by PCR (mean ± SEM, *n* = 3 biological replicates). **g** Relative enrichment of *CDK6* mRNA by LRPPRC antibody in the presence of ssDNA R14 or RTT (mean ± SEM, *n* = 3 biological replicates). **h** Quantification of *CDK6* mRNA in in different A549 cell colonies (mean ± SEM, *n* = 4 biological replicates). **i** Representative quantified *CDK6* mRNA degradation rate in different A549 cells colonies (mean ± SEM, *n* = 4 technical replicates). **j** Normalized G0 and G1 cell percentage in different A549 cell colonies after nocodozol was added (mean ± SEM.; *n* = 3 biological replicates). The Flow cytometry gating strategy was shown in supplemental Figure S10a. **k, l** Representative EDU staining images of different A549 cell colonies. The percentage of EDU-positive cells was quantified by Flow cytometry (mean ± SEM.; *n* = 3 biological replicates). The Flow cytometry gating strategy was shown in supplemental Figure S10c. **m** Representative quantified *CDK6* mRNA degradation rate in A549 cells before and after GAA (5 μM) treatment (mean ± SEM, *n* = 4 technical replicates). **n** Quantification of the *CDK6* mRNA in cells treated with GAA for 48 hours (mean ± SEM, *n* = 4 biological replicates). **o, p** Flow cytometry analysis of PI or EDU staining of LUAD cells treated with GAA, ribociclib (Ribo), or combination (Combi) (mean ± SEM.; *n* = 3 biological replicates). Immunoblotting results in **a**, EDU staining images in **k**, and the PCR results in images **i** and **m** were representative results of *n* = 3 independent experiments with similar tendency. Statistical significance in figures **f, g, h, l, n, o**, and **p** was determined by a one-way ANOVA analysis. Source data are provided as a Source Data file.

Since GAA, as a LRPPRC degrader, was screened out with the function of suppressing CDK6 expression (Fig. 1d), we explored whether LRPPRC could regulate CDK6 expression as well as its molecular mechanisms. Immunoblotting of A549 cells showed that *LRPPRC*^+/-^ cells expressed a downregulated protein level of CDK6, and the protein level of CDK6 decreased to an undetectable level in *LRPPRC*^-/-^ cells (Fig. 3a). Similar results were obtained with two more LRPPRC stable knockdown cell models (Supplementary Fig. S5a). In the hybridization-based mRNA expression array, we found *CDK6* mRNA had a 2-fold downregulation after LRPPRC genetic inhibition (Fig. 3b). In contrast, the expression of CDK4 and CDK2, another two proteins with similar functions as CDK6 in G1/S transition, did not show a sharp decrease after LRPPRC genetic inhibition, neither at the protein level detected by immunoblotting nor RNA level detected by mRNA array (Fig. 3a, b). These results indicated that LRPPRC could specifically regulate CDK6 expression, with minor effect on CDK2 and CDK4.

LRPPRC is an RNA-binding protein localized in both mitochondria and cytoplasm (Supplementary Fig. S5b, c), and mainly controls the stability of its binding RNAs^33–35^. The RNA binding protein immunoprecipitation sequencing (RIP-Seq) revealed 1441 potential binding RNAs of LRPPRC, which were highly reproducible with the two biological replicates (FPKM > 30, Fig. 3c, d, Supplementary Data 2, and Supplementary Fig. S5d-f ). KEGG enrichment analysis showed the LRPPRC-binding transcripts were primarily enriched in the cell cycle pathway (Supplementary Fig. S5g), and *CDK6* ranked the top among all the cell cycle-associated mRNAs, with a high RIP enrichment signal (RIP signal /input signal, Fig. 3d, e). Real-time PCR showed a RIP enrichment signal of about 500 for *CDK6*, which was much higher than that of *CDK2*, *CDK4*, and *CDK1*, indicating the high specificity and affinity between the interaction of LRPPRC and *CDK6* (Fig. 3f ). Furthermore, we found it was the C-terminus of LRPPRC that mediated the LRPPRC-*CDK6* interaction. 1 μM R14 (a ssDNA aptamer that specifically binds to the C-terminus of LRPPRC^28^) reduced the LRPPRC antibody-enriched *CDK6* by about 90%. In contrast, the control ssDNA sequence RTT did not noticeably affect the *CDK6* mRNA enrichment, even at a concentration of 10 μM (Fig. 3g). Real-time PCR results showed that the mRNA level of *CDK6* was significantly down-regulated in LUAD cells after LRPPRC knockout or knockdown (Fig. 3h and Supplementary Fig. S5h). In addition, the Actinomycin D (ACTD) based RNA stability experiment showed that LRPPRC knockout or knockdown shortened the half-life of *CDK6* (Fig. 3i and Supplementary Fig. S5i). Therefore, LRPPRC directly interacted with *CDK6* mRNA, enhanced *CDK6* stability, thus controlled the protein level of CDK6.

As a consequence of CDK6 expression inhibition, LRPPRC knockout significantly extended the time required for cells to bypass the G1/S checkpoint (Fig. 3j and Supplementary Fig. S6a, b) and reduced the proportion of cells incorporated with 5-ethynyl-2’-deoxyuridine (EDU) in unit time (Fig. 3k, l). This indicated LRPPRC was necessary for cell cycle G1/S transition. The roles of LRPPRC in cell cycle G1/S transition were further confirmed by analyzing the transcriptome data in The Cancer Genome Atlas Program (TCGA) database and the Avana dependency map dataset (DepMap, https://depmap.org/). The mRNA level of *LRPPRC* was positively correlated with cycle-associated gene sets (Cell Cycle, DNA Replication, et al.) and markers of cell cycle G1/S transition (Supplementary Fig. S6c-e). Meanwhile, cell proliferation was highly dependent on LRPPRC expression for most cell lines (Supplementary Fig. S6f, g). On the other hand, GAA treatment reduced the half-life time of *CDK6* mRNA and caused a decrease of CDK6 at both mRNA and protein levels (Fig. 3m, n). GAA treatment increased the percentage of cells in G0 and G1 phases, and decreased the percentages of EDU-incorporated cells (Fig. 3o, p). Furthermore, the combination of GAA and CDK4/6i showed a significant synergy effect in inducing G1/S arresting and reducing EDU incorporation (Fig. 3o, p). These results suggested that LRPPRC regulated CDK6 expression in a post-transcription-dependent manner. LRPPRC inhibition by GAA decreased CDK6 protein expression and induced G1/S arrest synergistically with CDK4/6i.

### CDK6 and E2F1 promoted LRPPRC expression, forming a positive feedback loop

**Fig. 4:**
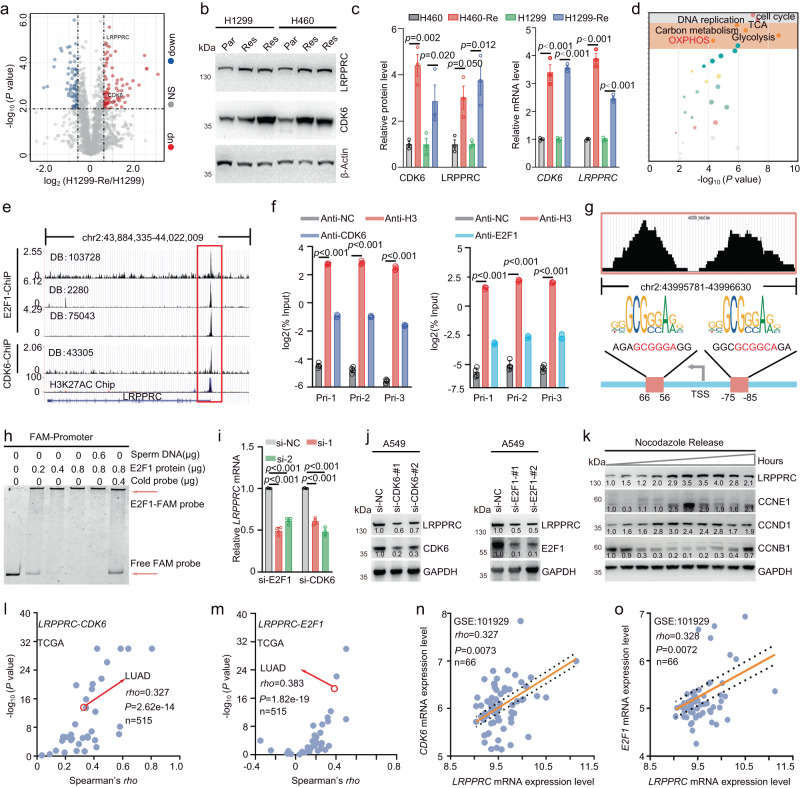
CDK6 and E2F1 promoted LRPPRC expression by enhancing transcription. **a** Volcano plot showed the quantitative dynamics of proteins in H1299 and H1299-Re quantified by non-labeled MS (*n* = 3 biological replicates). **b **Immunoblotting of indicated protein in parental cells (Par) and acquired-resistant cells (Res). **c** Quantification of protein and mRNA level of CDK6 and LRPPRC in parental and acquired-resistant cells (mean ± SEM*, n* = 3 biological replicates). **d** KEGG Pathway enrichment (performed using KOBAS software) of differentially expressed proteins in H1299 and H1299-Re. **e** Mapping of CDK6, E2F1, or H3K27AC Chip-seq reads back to the genomic locus of *LRPPRC*. The figure was obtained from Cistrome database. **f** Quantification of *LRPPRC* promoter in Chip products using CDK6 antibody, E2F1 antibody, histone H3 antibody (Anti-H3, positive control), and non-immune antibody (Anti-NC, negative control) in A549 cells (mean ± SEM, *n* = 4 biological replicates). **g** Diagram of the binding sites in *LRPPRC* promoter interacted with E2F1 protein. The conserved E2F1 binding sequences were also shown. **h** EMSA experiment testing the interaction between E2F1 and *LRPPRC* promoter. **i** Quantification of *LRPPRC* mRNA in A549 cell before and after knockdown of either CDK6 or E2F1 (mean ± SEM, *n* = 4 biological replicates). **j, k** Immunoblotting of LRPPRC in A549 cells transfected with indicated siRNAs or released from nocodazole treatment at different time points. **l, m** Correlation analysis of indicated mRNAs in different cancer types. The correlation score, sample number, and *P* value were obtained from the online analysis website TIMER (https://cistrome.shinyapps.io/timer/). **n, o** Correlation analysis of indicated mRNAs in LUAD samples provided by the GEO dataset (GSE101929). The Immunoblotting results in figure **b, j**, and **k** were representative results of *n* = 3 independent experiments with the same tendency. The statistical significance of results in figures **c, f,** and **i** was determined by a one-way ANOVA analysis. The statistical significance of the correlation analysis in figure **l-o** was determined by a two-tailed nonparametric Spearman correlation analysis. Source data are provided as a Source Data file.

According to our screening results, GAA reversed the resistance of CDK4/6i in acquired resistance cell models (Fig. 1g and Supplementary Fig. S3g), which indicated that LRPPRC could also play an essential role in acquired resistance. We thus checked the changes of LRPPRC protein itself before and after acquired CDK4/6i resistance. Proteomics study was carried out with CDK4/6i sensitive cell H1299 and corresponding acquired drug-resistant cell H1299-Re. A total of 212 proteins were differentially expressed between H1299-Re and H1299 cells (fold change>1.5 or <0.5, *P* value < 0.05, Supplementary Data 3), including the most studied CDK6 protein. Surprisingly, we found the mass spectrum (MS) score of LRPPRC protein was also significantly upregulated in H1299-Re cells, with a *P-*value ranking third among all differentially expressed proteins (Fig. 4a). Immunoblotting and PCR quantification further confirmed the changes of LRPPRC and CDK6, both showed a 2-4 fold increase after cells acquired CDK4/6i resistance (Fig. 4b, c).

Besides LRPPRC and CDK6 overexpression, KEGG pathway analysis found that the differentially expressed proteins were primarily enriched in mitochondrial metabolism, the most well-studied downstream targets of LRPPRC (Supplementary Data 4)^36^. It is known that LRPPRC maintains cell OXPHOS by binding to the mRNAs of the mitochondrial genome-encoded OXPHOS complex subunits^35^. The MS scores of critical subunits of OXPHOS and TCA were significantly increased in H1299-Re cells, and protein components involved in glycolysis decreased after cells acquired CDK4/6i resistance, which were further confirmed by immunoblotting and PCR quantification (Fig. 4d and Supplementary Fig. S7a-c). The oxygen consumption rate (OCR) experiment showed that the acquired-resistant cells harbored significantly higher values of basal respiration, maximum respiratory potential, spare respiratory capacity, and mitochondrial ATP yield ability than the corresponding parent cells (Supplementary Fig. S7d, e). All these results demonstrated that LRPPRC and its downstream proteins were enhanced during CDK4/6i treatment.

Then, we focused on transcriptional regulation to study the molecular mechanism for the enhanced LRPPRC expression in response to CDK4/6i, as both mRNA and protein levels of LRPPRC were significantly upregulated after CDK4/6i treatment. We selected CDK6 and E2F1 as potential LRPPRC regulators for a list of reasons. Elevated expression of CDK6 was the most significant and universal feature during CDK4/6i treatment, which ensured CDK6 and its downstream target E2F1 effectively perform the transcriptional activity in the context of CDK4/6i treatment. E2F1 is a classical transcription factor, and CDK6 is involved in the transcriptional regulation of gene expression in a kinase-independent manner^17, 18,37^, all of which have been reported in promoting cell mitochondrial metabolism, including OXPHOS^38,39^. By analyzing Chip-seq data in public databases Cistrome (http://www.cistrome.org/), we found that CDK6 and E2F1 bound to the upstream sequence of the *LRPPRC* gene in different datasets, with a binding score ranking in the top 30% of all transcription factors (Supplementary Fig. S7f ). Furthermore, the binding positions of the two proteins on the *LRPPRC* gene were highly overlapped (Fig. 4e). At last, the binding sites of CDK6 and E2F1 on the *LRPPRC* gene were also the region enriched for histone acetylation modification (characteristics of the promoter region, Fig. 4e).

Chip-PCR confirmed the binding of CDK6 and E2F1 on the *LRPPRC* promoter. Compared with the negative control antibody, both CDK6 and E2F1 antibodies can enrich 8-10-fold *LRPPRC* promoter sequence (Fig. 4f ). Considering that CDK6 or the CDK6-CCND1 complex does not have DNA-binding function, we expected that CDK6 binds to LRPPRC promoter indirectly^17,18^. For E2F1, sequence analysis revealed two conserved E2F1 protein-binding motifs in the promoter region of *LRPPRC* (Fig. 4g). Electrophoretic mobility shift assay (EMSA) showed FAM-labeled *LRPPRC* promoter DNA could interact with purified E2F1 protein directly, which could be disrupted by cold probes without FAM (Fig. 4h). Knockdown of either CDK6 or E2F1 with specific siRNAs lowered the protein and RNA level of *LRPPRC* effectively (Fig. 4i, j and Supplementary Fig. S7g, h). In addition, nocodozol-assisted cell cycle synchronization showed a cell cycle-dependent expression of LRPPRC protein, and the expression pattern of LRPPRC was consistent with the pattern of CDK6-CCND complex expression and the transcriptional activity of E2F1 during the cell cycle. More specifically, the protein level of LRPPRC started to increase when cells entered the G1 phase (the marker is CCND1 expression, where the activity of CDK6-CCND1 and E2F1 began to increase). The LRPPRC level peaked after cells by passing the G1/S checkpoint, where CCND1 and CCNE1 reached the highest level. The LRPPRC protein started to decline after the cells entered into the division phase (the marker is CCNB1 expression) (Fig. 4k). The above experiments confirmed that CDK6 and E2F1 could promote the expression of LRPPRC by binding to the promoter of *LRPPRC*.

At last, to further support the regulation between LRPPRC-CDK6 and E2F1, we analyzed the correlation of the RNA levels of these three genes in clinical samples. Transcriptome data from the TCGA database showed that the RNA level of *LRPPRC* was positively correlated with that of *CDK6* (n = 515, *Rho* = 0.327, *P* < 0.0001) and *E2F1* (n = 515, *Rho* = 0.383, *P* < 0.0001) in lung adenocarcinoma; and the positive correlation tendency was widespread in different tumors (Fig. 4l, m and Supplementary Fig. S7i). We also verified this correlation in GEO datasets and found the expression profile data from different research groups also supported the positive correlation between *LRPPRC* and *CDK6* and *E2F1* (Fig. 4n, o and Supplementary Fig. S7j, k).

Therefore, while LRPPRC stabilized *CDK6* mRNA, CDK6 protein and its downstream protein E2F1 could also act as transcription-associated factors to enhance the expression of LRPPRC, thus forming a positive feedback loop.

### LRPPRC-CDK6 loop inhibition also suppressed OXPHOS and eliminated cancer stem cells

The above results have elucidated the roles and mechanisms of LRPPRC in regulating CDK6 expression and CDK4/6i sensitivity. We transfected CDK6 overexpression plasmid into LRPPRC stable knockdown cells (Supplementary Fig. S8a). Cell colony-forming assays found that re-expression of CDK6 in LRPPRC knockdown cells could only partially restore its resistance against CDK4/6i (Supplementary Fig. S8b). These results indicated that LRPPRC was also involved in regulating CDK4/6i sensitivity, beyond controlling CDK6 expression. The most studied functions of LRPPRC are promoting OXPHOS and cancer stem cells (CSCs)^24,40^, both of which have been shown to promote CDK4/6i resistance in some cancer types other than LUAD, such as glioma and bladder cancer^41–44^. Our results showed significantly increased expression of LRPPRC and OXPHOS after acquired CDK4/6i resistance in LUAD cells (Fig. 4a-d). We then asked if the classical functions of LRPPRC, such as OXPHOS and CSC maintenance, were also involved in CDK4/6i response in LUAD, which could also be suppressed by GAA.

**Fig. 5:**
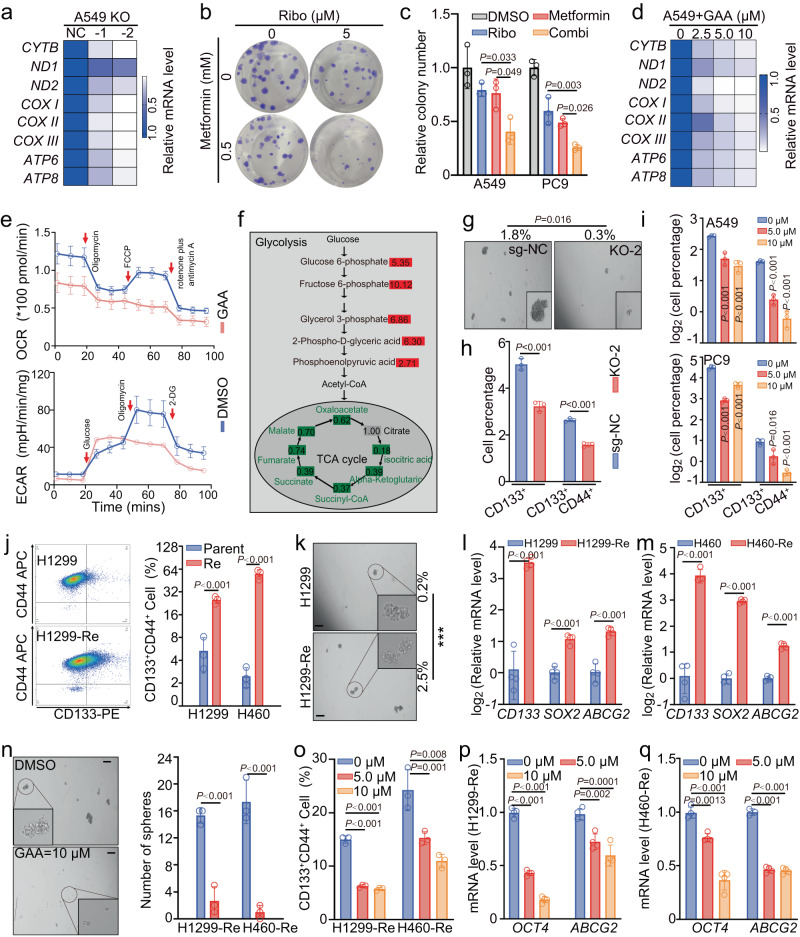
LRPPRC-CDK6 inhibition suppressed OXPHOS and eliminated cancer stem cells. **a** Heatmap of relative abundance of indicated mRNAs in A549 cells before and after LRPPRC knockout. **b, c** Representative images and quantification of colony formation of A549 cells treated with metformin, ribociclib (Ribo), or combination (Combi) (mean ± SEM.; *n* = 3 biological replicates). **d** Heatmap of relative abundance of indicated mRNAs in A549 cells treated with GAA. **e** OCR (mean ± SEM.; *n* = 5 biological replicates) and ECAR (mean ± SEM.; *n* = 4 biological replicates) analysis of A549 cells before and after GAA treatment. **f** Overview of changes in central carbon metabolism after GAA treatment. Metabolite changes were given as folds of control (mean ± SEM.; *n* = 3 biological replicates). **g** Representative images and number quantification of tumorspheres of A549 before and after LRPPRC knockout (mean ± SEM.; *n* = 3 biological replicates). **h, i** Quantification of CD133^+^, or CD44^+^CD133^+^ cells in cells with LRPPRC knockout or GAA treatment (mean ± SEM.; *n*= 3 biological replicates). **j** Representative flow cytometry images of parental cells (H1299) and acquired CDK4/6i-resistant cells (H1299-Re). The percentage of CD44^+^CD133^+^ cells was quantified (mean ± SEM.; n = 3 biological replicates). **k** Representative images and number quantification of tumorspheres of parental and acquired CDK4/6i-resistant cells (mean ± SEM.; *n* = 3 biological replicates). **l, m** Quantification of CSC mRNA markers in parental and acquired resistant cells (mean ± SEM, *n* = 4 biological replicates). **n** Representative images of tumorspheres generated from H1299-Re cells treated with DMSO or GAA. The number of tumorspheres per 1000 cells was quantified (mean ± SEM.; *n* = 3 biological replicates). **o** Quantification of CD44^+^CD133^+^ cells in H1299-Re cells treated with different concentrations of GAA (mean ± SEM.; *n* = 3 biological replicates). **p, q** Quantification of CSC mRNA markers in acquired resistant cells treated with different concentrations of GAA (mean ± SEM, *n* = 3 biological replicates). The Flow cytometry gating strategy in figure **h, i**, **j**, and **o** was shown in supplemental Figure S10d. The statistical significance of quantification results in figure **c, i, o, p,** and **q** was determined by a one-way ANOVA analysis. Statistical significance in figure **h, j, l, m**, and **n** was determined by a two-tailed unpaired Student’s *t*-test. Source data are provided as a Source Data file.

In the primary resistance A549 cells, LRPPRC knockout significantly reduced the mRNA level of OXPHOS subunits encoded by mtDNA, consistent with published works by other groups^21,25^ (Fig. 5a). We tested the combination of CDK4/6i with the metformin, the most studied OXPHOS inhibitor. The colony-forming assays showed this combination harbored significantly better anti-tumor activity than the single drug, indicating the LRPPRC-mediated OXPHOS could also promote CDK4/6i resistance in LUAD (Fig. 5b, c). LRPPRC inhibition by GAA effectively reduced the expression of OXPHOS complex subunits at both RNA and protein levels (Fig. 5d and Supplementary Figure S8c). The OCR experiment showed that the maximum respiratory potential, spare respiratory capacity, and coupling efficiency were all significantly inhibited by GAA (Fig. 5e and Supplementary Figure S8d). In the extracellular acidification rate (ECAR) assay, the GAA-pretreated cells showed a more significant increase in ECAR after glucose addition, indicating more glucose went to glycolytic metabolism (Fig. 5e and Supplementary Figure S8d). Liquid chromatography–mass spectrometry (LC-MS) was applied to check the change of central carbon metabolites after LRPPRC inhibition by GAA. Intermediates belonging to TCA cycle were broadly downgraded. In contrast, the steady-state levels of most intermediates belonging to glycolysis steps were increased with high multiples (Fig. 5f and Supplementary Data 5). Similar to the results from primary resistant cells, GAA treatment reduced the mRNA and protein level of OXPHOS subunits encoded by mtDNA (Supplementary Figure S8e, f), as well as the maximum respiratory potential and coupling efficiency (Supplementary Figure S8g-i) in acquired-resistant cells. Therefore, LRPPRC-regulated OXPHOS promoted CDK4/6i resistance, which could also be suppressed by GAA treatment.

Tumor stem cells are another major driver of drug tolerance and treatment failure. We also asked whether LRPPRC-CDK6 inhibition could further eliminate tumor stem cells. Flow cytometry analysis showed a significant decrease in lung cancer CSC marker CD133 after LRPPRC knockout (Fig. 5g, h)^45,46^. In vivo tumorigenic experiments showed that *LRPPRC*^-/-^ cell completely lost the tumorigenic ability, indicating LRPPRC was necessary for lung cancer stem cells maintainance (Supplementary Fig. S8j). GAA treatment gradually reduced the percentage of CD133^+^ cells, and the number of tumorspheres formed in suspension culture (Fig. 5i and Supplementary Fig. S8k, l). For the acquired resistant cells, flow cytometry showed a higher proportion of CD133^+^ CSCs, and more tumorspheres formed by suspension culture than parental cells (Fig. 5j, k). Furthermore, PCR quantification showed that the mRNA levels of CSC markers were upregulated remarkably in acquired-resistant cells (Fig. 5l, m). These results suggested the acquired-resistant cells harbored significantly more CSCs. Similar to the results from primary resistant cells, GAA treatment was also effective in reducing the percentage of CD133^+^CD44^+^ cells, mRNA levels of the CSC-specific transcription factors, and the number of tumor spheroids for these acquired-resistant cells (Fig. 5n-q).

Taken together, the activation of the LRPPRC-CDK6 positive feedback loop also stimulated OXPHOS and CSC generation, thus further promoting CDK4/6i resistance. LRPPRC-CDK6 loop inhibition by GAA not only induced cell cycle arrest by decreasing CDK6 expression, but also suppressed OXPHOS and eliminated cancer stem cells.

### LRPPRC-CDK6 loop inhibition increased CDK4/6i sensitivity in vivo

**Fig. 6:**
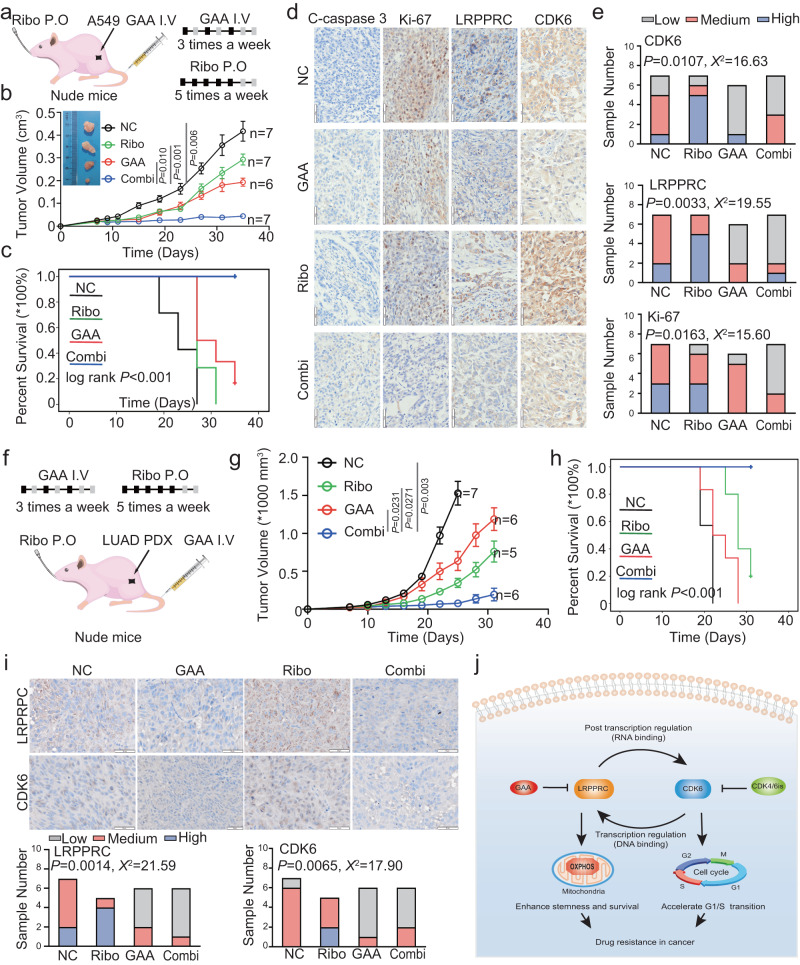
GAA inhibited LRPPRC-CDK6 feedback loop and increased CDK4/6i sensitivity in vivo. **a** Schematic diagram of GAA treatment in mice bearing A549 cells generated subcutaneous tumor. **b** Tumor volume of subcutaneous A549 cells treated with indicated drugs (mean ± SEM.; *n* = 1 experiment; *n* = 6 mice in GAA treatment group (GAA) and *n* = 7 mice in other groups). **c** Kaplan–Meier simulated survival analysis of mice inoculated with A549 cells and treated with indicated compounds. **d, e** IHC images and quantification of IHC intensity of indicated proteins in A549 cells xenografts treated with different drugs. **f** Schematic diagram of GAA treatment with mice model bearing subcutaneous tumor generated from clinical LUAD samples. **g** Tumor volume of subcutaneous LUAD tissues in the mice treated with indicated drugs (mean ± SEM.; *n* = 1 experiment; *n* = 7 mice in the control group (NC)*, n* = 6 mice in GAA treatment group (GAA), *n* = 5 mice in ribociclib treatment group (Ribo), and n = 6 mice in the combination treatment group (Combi)). **h** Kaplan–Meier simulated survival analysis of mice inoculated with LUAD tissues and treated with indicated compounds. **i** IHC images and quantification of IHC intensity of indicated proteins in PDX xenografts treated with different drugs. The statistical significance of quantification results in figure **b** was determined by a two-way ANOVA analysis for comparison at the endpoint. IHC images **d** and **i** were representative results of *n* = 3 independent experiments. The statistical significance of quantification results in figure **g** was determined by a two-way ANOVA analysis for comparison at day 25. The statistical significance of quantification results in figure **c** and **h** was determined by Kaplan-Meier analysis. Statistical significance of quantification results in figure **e** and **i** was determined by a Chi-square test. Source data are provided as a Source Data file. **j** Schematic diagram of the principle of this work. LRPPRC binds to *CDK6* mRNA and promotes CDK6 expression. CDK6 and its downstream E2F1 bind to the promoter of LRPPRC and promote its expression, forming a positive feedback loop. The combination of CDK4/6i and LRPPRC inhibitor GAA arrests cells in the G0/G1 phase and eliminates tumor stem cells.

At last, we investigated if LRPPRC-CDK6 loop inhibition could increase CDK4/6i sensitivity in vivo. Two LUAD mice models were used, including subcutaneous tumors formed by A549 cells and a PDX model derived from LUAD patient tissues. In the first subcutaneous tumor model, A549 cells were injected subcutaneously, and mice were divided into four groups: control group, GAA treatment group, ribociclib treatment group, and combination treatment group. Ribociclib was suspended in cyclodextrin solution and administered by intragastric administration. GAA was administered by tail vein injection in the form of nanoliposome, which can increase the effectiveness of GAA on tumors (Fig. 6a). The results of electron microscopy and Nano flow analysis showed that GAA-nanoliposome had been successfully constructed, with a diameter of about 71 nm (Supplementary Fig. S9a-c). After four weeks, both single treatments by GAA or CDK4/6 inhibitor ribociclib only partially delayed the growth of subcutaneous tumors, while the combination of the two drugs almost completely inhibited the tumor proliferation. The volume of subcutaneous tumors in the combined treatment group was significantly smaller compared to the other groups (Fig. 6b). The simulated survival curve also showed that the drug combination effectively prolonged the survival time of mice (Fig. 6c).

To confirm that this combination suppressed the LRPPRC-CDK6 feedback loop in vivo, we prepared tumor tissue sections from A549 cell subcutaneous xenograft and performed IHC assay. Tumor tissues from CDK4/6i monotherapy showed higher protein levels of LRPPRC and CDK6 compared to the control group, indicating the activation of the LRPPRC-CDK6 feedback loop after CDK4/6i treatment in vivo. However, the tumor tissues in the GAA treatment group and the combination group showed lower levels of LRPPRC and CDK6 than the control group and CDK4/6i monotherapy group, suggesting that the addition of GAA can also block the LRPPRC-CDK6 feedback loop in vivo. Furthermore, tissue sections in the combination group showed the weaker staining intensity of cell proliferation marker Ki-67 than other groups, while the staining intensity of apoptosis marker cleaved-caspase 3 did not show any difference among all the groups. This indicated the combination of GAA and ribociclib mainly induced cell cycle arrest, rather than apoptosis, consistent with the results observed in vitro (Fig. 6d, e).

Besides the LUAD models derived from cell lines, we also constructed lung adenocarcinoma PDX models, which are more similar to human tumors’ actual characteristics for drug efficacy testing. We selected PDX model LP2, with the highest LRPPRC protein level, from six successfully constructed PDX models for subsequent drug administration (Supplementary Fig. S9d). The experimental grouping and drug administration design were consistent with the A549 subcutaneous xenograft experiment described above (Fig. 6f). For monotherapy of CDK4/6i, the PDX model showed good sensitivity in the first week of administration but subsequently displayed a rapid tumor volume increase, suggesting that the tumor had acquired drug resistance. Combining the two drugs resulted in a more robust reduction in tumor volume and a significantly extended median survival time compared to either ribociclib or GAA treatment alone (Fig. 6g, h). Nevertheless, the body weight of the mice in the combination group did not show a significant difference with single drug treatment groups, indicating that such a combination strategy was well tolerated (Supplementary Fig. S9e). Further IHC results of both CDK6 and LRPPRC in the PDX model were similar to that observed in the subcutaneous xenograft experiment (Fig. 6i).

The in vivo drug efficacy evaluations and molecular analysis confirmed that the combination of GAA and CDK4/6i effectively suppressed the LRPPRC-CDK6 feedback loop and increased the therapeutic efficacy of CDK4/6i.

## Discussion

CDK4/6i has broad application prospects for the targeted therapy of solid tumors. However, drug resistance caused by abnormal expression of CDK6 is a major obstacle to the clinical application of CDK4/6i. Exploring the factors responsible for CDK6 up-regulation in resistance development and designing intervention strategies are essential for the clinical use of CDK4/6i. This work has demonstrated a druggable LRPPRC-CDK6 positive feedback loop responsible for CDK6 expression and CDK4/6i response, as well as a drug combination strategy to overcome this positive feedback loop. The combination of CDK4/6i with LRPPRC inhibitor GAA significantly improves the therapeutic effect of CDK4/6i in LUAD (Fig. 6j). Considering the overexpression of LRPPRC in multiple tumor types, our drug combination strategy has the potential to be effective in various solid tumors.

Although the importance of overexpressed CDK6 protein for CDK4/6i resistance has been widely recognized, the molecular mechanism contributing to CDK6 protein up-regulation and the pathways where CDK6 promotes CDK4/6i resistance remain poorly understood. We have unveiled the LRPPRC-mediated post-transcriptional regulation of CDK6 protein expression. It is worth mentioning, we performed whole-exome sequencing on H1299 and H1299Re cells (Sequence Read Archive (SRA) repository, PRJNA953351), and found that the acquired-resistant cells did not show variation on CDK6 gene, or genes that have been reported to affect CDK6 expression such as FAT Atypical Cadherin 1 (FAT1). The results further confirmed the elevated CDK6 by transcriptional regulation or post-transcriptional regulation, such as LRPPRC-CDK6 loop. Moreover, we find that CDK6 can promote the expression of onco-protein LRPPRC via transcriptional regulation, thereby promoting OXPHOS and stem cell maintenance. Both OXPHOS and cancer stem cells have been reported to be essential factors for the failure and recurrence of CDK4/6i therapy. Our research provides a reference for understanding the molecular regulation between CDK6, cell metabolism, and CSC generation. As we have demonstrated that the use of LRPPRC-specific inhibitor, GAA, reduces CDK6 expression, we offer a approach for therapy against cancer stem cells by targeting LRPPRC.

GAA, as an old medicine in gynecology, has shown tumor-suppressive effects and has been introduced in clinical trials for lung cancer and other cancers. In these clinical trials, GAA has been regarded as a BCL2 inhibitor when combined with chemotherapy drugs for unscreened patients. However, the overall clinical benefit remains modest^47–49^. Based on our previous work reporting GAA as an LRPPRC inhibitor, we further clarified that LRPPRC, rather than BCL2, is the priority target of GAA in CDK4/6i treatment. According to our results, the combination of GAA and CDK4/6i induced cell cycle G1 arrest, but not apoptosis. We expected that the prolonged G1 arrest could prevent cells to complete DNA replication and exit the cell cycle, or further induce irreversible cell senescence^50,51^. Although the mechanisms underlying the observed cell growth inhibition still need further investigation, this work offers valuable information for identifying beneficial patients for GAA based precision therapy. Of course, we must also admit that GAA is only a primary candidate drug of LRPPRC, still facing problems such as limited affinity and relatively high effective concentration. Given the important roles of LRPPRC in CDK4/6i sensitivity, developing LRPPRC inhibitors will have important clinical prospects.

## Methods

All mouse experiments were approved by the Institutional Animal Care and Use Committee of Zhejiang Cancer Hospital and carried out following their legal requirements. Approval for using the clinical samples was obtained from the institutional ethics committee of Zhejiang Cancer Hospital (Ethical number: IRB-2020-431).

### Cell culture and treatment

HEK-293FT (1101HUM-PUMC000364), H1944 (3101HUMSCSP596), H1299 (1101HUM-PUMC000469), A549 (1101HUM-PUMC000002), H460 (4201HUM-CCTCC00109) and H23 (4201HUM-CCTCC00651) were purchased from Chinese National Infrastructure of Cell Line Resource (Beijing, China). The PC9 cell line is a gift from professor Yongmei Song (National Cancer Center/ Cancer Hospital, Chinese Academy of Medical Sciences and Peking Union Medical College, China). All cell lines used in this study are free of *mycoplasma* contamination and have been authenticated before the start of experiments using STR DNA fingerprinting. All LUAD cells were cultured in RPMI-1640 medium, and HEK-293FT cells were cultured in DMEM medium, with 10% FBS (#10099141 C, Gibco, America). To generate *LRPPRC*^+/-^ and *LRPPRC*^-/-^ cell lines, *LRPPRC*-specific single-guide RNA (sgRNA) was cloned into the pLentiCRISPR-E plasmid (#78852, Addgene, America). HEK-293FT cells in a 6-cm dish were transfected with 4 μg pLentiCRISPR-E vector containing LRPPRC-specific sgRNA and lentiviral packaging plasmids psPAX2 (2 μg), pMD2.G (2 μg) at a confluence of about 90%. The medium was replaced by 3 mL fresh DMEM medium containing 20% FBS 6 hours later. 48 hours After transfection, cell medium containing lentivirus was harvested and filtered through a 0.45-μm filter. A549 cells were then infected by the lentivirus at ~70% confluence, followed by selection with 2 μg/mL puromycin for 72 hours. Survival cells were seeded into 96-well plates to obtain single cell-derived clones. Immunoblotting was used to confirm the knockout efficiency of LRPPRC in single-cell-derived clones. To generate LRPPRC stable knockdown cells, lentivirus containing LRPPRC-specific shRNA was purchased from Genechem Company (Shanghai, China). Cells were infected with diluted lentivirus for 48 hours, and then the puromycin-containing medium was added to select the successfully infected cells. The information of all sgRNA and shRNA sequences we used were listed in Supplementary Data 1.

### Screening molecules harboring synergy effect with CDK4/6i

The natural product library was purchased from Thermo Fisher and diluted into a 10 μM stock solution by the complete medium. PC9 and A549 cells were seeded in 96-well plates at a density of 2,000 cells/well and incubated overnight. Each molecular probe was added alone (final concentration of 5 μM) or with ribociclib (#MB5136, Meilunbio, China) together (final concentration: prober 5 μM; ribociclib 4 μM). After 96 hours of treatment, cell viability was detected and quantified by the CCK8 kits (#C0005, Topscience, China). According to the absorbance of CCK8 of each group, synergistic or antagonistic effects were determined by calculating the coefficient of drug interaction (CDI, CDI = AB/(A × B). A or B is the ratio of the CCK8 signal of single drug group to the control group, AB is the ratio of the CCK8 signal of the two-drug combination group to the control group. CDI < 1 indicates synergism, CDI = 1 indicates additivity and CD > 1 indicates antagonism.

### Colony formation and cell proliferation assay

Briefly, cells were seeded in plates at a density of 300 cells/well, and drugs were added 24 h after cell plating. Cells were exposed to drugs or solvent for 1–2 weeks, with the medium changed and fresh drug added every 3 days. Cells were fixed with ethanol and stained with 1% crystal violet solution.

Cell proliferation experiments were carried out using the xCElligence RTCA MP (Agilent, America). Cells were seeded into electrodes coated 16-well plates at a density of 2000/well. Drugs were added about 24 hours after cell inoculation. The numbers of adherent cells were measured every 1 hour and displayed as cell index or normalized cell index. Cell proliferation curves were obtained based on the cell index data at different time points.

### Generation of cells with acquired resistance against CDK4/6i

Cell lines with acquired resistance against CDK4/6i ribociclib were derived by treating cells with increasing concentrations of ribociclib starting at 500 nM. Cells were first cultured with 500 nM ribociclib for one week, then cultured with 1 μM ribociclib for four weeks, 5 μM, 10 μM, 15 μM, and 20 μM ribociclib for 6 weeks sequentially and respectively. Cell lines with acquired resistance derived from ribociclib treatment were maintained in 20 μM of ribociclib.

### Immunoblotting analysis

Cells were lysed in RIPA lysis buffer (#C1053-100, Applygen, China), containing protease inhibitor cocktail (#P1266-1, Applygen, China), and phosphatase inhibitors (#P1260-1, Applygen, China). Equal amounts of protein (30-60 μg) were separated by 10% SDS–PAGE and transferred to the PVDF membrane (#03010040001, MERCK, America). The primary antibodies were diluted with BSA using the following concentrations: Anti-GAPDH (Cell Signaling Technology, #97166, 1:5000), Anti-LRPPRC (Santa Cruz Biotechnology, #sc-166178, 1:2000), Anti-P-RB1 Ser807/811 (Cell Signaling Technology, #8516, 1:500), Anti-P-RB1 Ser780 (Cell Signaling Technology, #8180, 1:500), Anti-total OXPHOS antibody cocktail (Abcam, ab110413, 1:2000. This cocktail contains 5 mouse mAbs anti-NDUFB8, anti-SDHB, anti-COX1, anti-UQCRC2 and anti-ATP5A), Anti-CDK6 (Cell Signaling Technology, #3136, 1:500), Anti-CDK4 (Cell Signaling Technology, #12790, 1:500), Anti-CDK2 (Cell Signaling Technology, #18048, 1:500), Anti-RB1 (Cell Signaling Technology, #9313, 1:500), Anti-E2F1 (Cell Signaling Technology, #3742, 1:500), Anti-CCNE1 (Cell Signaling Technology, #20808, 1:500), Anti-PARP1 (Cell Signaling Technology, #9542, 1:500), Anti-CASPASE 3 (Cell Signaling Technology, #9662, 1:500), Anti-BCL2 (Cell Signaling Technology, #4223, 1:500), Anti-CCNB1 (Cell Signaling Technology, #12231, 1:1000), Anti-PCNA (Proteintech, 10205-2-AP, 1:1000), Anti-α/β-Tubulin (Cell Signaling Technology, #2148, 1:5000), Anti-β-Actin (Cell Signaling Technology, #3700, 1:10000). After thorough washing, the HRP-labeled secondary antibody (PROMEGA, W401B, 1:3000; W402B, 1:2000) was added for 1 hour at room temperature. The protein luminescence signal was acquired using Amersham ImageQuant 800 (GE, America).

### RIP-seq and RIP-PCR

RNA immunoprecipitation was performed with the Magna RIP RNA-Binding Protein Immunoprecipitation Kit (#17-700, Millipore, Germany) according to the manufacturer’s instructions. Briefly, A549 cells were seeded in the 10-cm dishes and harvested at ~90% confluence. The cell lysates were incubated with 2 μg anti-LRPPRC antibody (Abcam, ab97505) or rabbit IgG control involved in the kit. Then the cell lysates were incubated with protein A/G modified beads for 4 h at 4°C in a rotator. Co-immunoprecipitated RNAs were extracted by a miRNeasy Mini Kit (#217004, QIAGEN, Germany) and subjected to RNA sequencing or PCR verification. The information of all PCR primers we used in the RIP experiment was listed in Supplementary Data 1. The RNA sequence was performed by the ECHO BIOTECH company (China). Briefly, a total of 10 ng RNA was subjected to sequencing library construction using the SMARTer Stranded Total RNA-Seq Kit V2 (Takara Bio USA, Inc.). Sequencing was performed on an Illumina NovaSeq platform, adapters on reads were removed using the software Cutadapt (version 1.18). Mapping was performed using the software HISAT2 (version 2.1.0) against the Human GRCh38(hg38) to get sam files. The Sam files were sorted by the software Samtools (version 1.6) view to get sorted Bam files. The mRNA expression level was quantified by the software Stringtie (version, 1.3.3b) to compare the sorted Bam files with human genes gff file (Ensembl release 101).

### Flow cytometry

For CD44 and CD133 staining, cells were collected by trypsin digestion and low-speed centrifugation (80 × g). 5 × 10^5^ cells were dispersed in 100 μL PBS and cocultured with PE-conjugated anti-human CD133 (#372804, Biolegend, America, 1:20) and APC-conjugated anti-human CD44 (#559942, BD Pharmingen, America, 1:40) for 30 minutes on ice and then analyzed by a flow cytometer cytoFLEX LX (Beckman, America). For cell cycle analysis, cells were fixed with pre-cooled 75% ethanol overnight. After washing by PBS, 0.5 mL staining solution (containing 50 μg/mL propidium iodide (#P4170, MERCK, America), 0.1% Triton-X-100, and 100 μg/mL RNase A (#RNASEA-RO, MERCK, America)) was added and stained for 30 min at 37°C. The staining signal of propidium iodide was analyzed by cytoFLEX LX (Beckman, America). To evaluate the speed of cells bypassing the G1/S checkpoint, nocodazole (#M1404, MERCK, America, 50 ng/mL) was added to prevent cells from entering the G1 phase from the G 2/M phase. Then, cells were harvested at different times after nocodazole was added and subjected to PI staining and cytoFLEX LX analysis. The EDU incorporation assay was performed using Yefluor 488 EdU Imaging Kits following the manufacturer’s instructions (#40278ES25, Yeasen, China). The green fluorescence was connected to the incorporated EDU by a click reaction, and the nucleus was calibrated in blue by DAPI (#D9542, MERCK, America). The green signal of EDU and the blue signal of the nucleus were further analyzed by flow cytometry (Beckman, America). For the Annexin V/PI staining assay, cells were harvested by 0.25% trypsin-EDTA and then washed thrice with cold PBS buffer. An Annexin V apoptosis detection Kit (#40302ES60, Yeasen Biotechnology, China) was used to detect Annexin V and PI staining signals. Briefly, cells were suspended in 200 μL binding buffer, then add 10 μL of FITC-conjugated Annexin V antibody for 15 min at room temperature. Five minutes before the flow cytometry detection, add 5 microliters of PI solution. The results generated from the above flow cytometry experiments were further analyzed by the software FlowJo (version 10).

### Sphere formation assay

Cells were collected and washed by PBS three times to remove serum. Then cells were suspended in serum-free RPMI-1640 medium (containing 20 ng/mL EGF (#abs04176, ABSIN, China), 20 ng/mL bFGF (#abs04181, ABSIN, China), and 2% B27 supplement (#15596026, Thermo Fisher Scientific, America)). Cells were subsequently cultured in ultra-low attachment 24-well plates at a density of 1000-5000 cells per well. After 1-2 weeks, the number of spheres (>10 cells/spheroid) was counted. The spheres were collected by low-speed centrifugation (80 × g) and then subjected to further experiments.

### Mitochondria fractionation analysis

Cells were harvested by 0.25% trypsin-EDTA dissociation and centrifugation. The mitochondria and cytoplasmic fractions were separated and extracted with a mitochondria isolation kit (#C1260, Applygen, China). After protein fractionation, 10 μg of mitochondrial or cytoplasmic protein were subjected to western blotting to test the fractionation purity. ATP5A was used as a marker for mitochondrial components, and β-Actin was used for cytoplasmic components.

### siRNA transfections, RNA isolation, cDNA synthesis, and qRT–PCR

The siRNAs were designed and synthesized by GenePharma (China). Cells were seeded in 6-well plates, and siRNAs were dissolved in DEPC water and introduced into cells using a lipofectamine 3000 kit (#L3000150, Thermo Fisher Scientific, America); the final siRNA concentration was 50 nM. 48 hours later, cells were harvested and subjected to RNA and protein detection. Total RNA was isolated from cells using TRIzol Reagent (#15596026, Thermo Fisher Scientific, America) following the manufacturer’s instructions. 2 μg of total RNA was subjected to first-strand cDNA synthesis using the Hifair II 1st Strand cDNA Synthesis Kit (#11111ES92, Yeasen, China). Briefly, DNase I was used to remove residual genomic DNA at 42 °C. And then, reverse transcriptase and random primers were added to synthesize cDNA at 55 °C. RT-PCR was performed in a 20-μL reaction volume according to the manufacturer’s protocol of SYBR Premix Ex Taq (#11195ES03, Yeasen, China). The sequences of all siRNAs and PCR primers we used were listed in Supplementary Data 1.

### Chip assay

The chip experiment was performed using SimpleChIP Enzymatic Chromatin IP Kit (#56383, Cell Signaling Technology, America) according to the manufacturer’s instructions. Briefly, a total of 2 × 10^6^ proliferating A549 cells were used for each immunoprecipitation reaction. Chromatin fragmentation was prepared by sonication by using a ME220 ultrasonic disruptor (COVARIS, America). Immunoprecipitation was performed using 5 µg of CDK6 antibody (Proteintech, 14052-1-AP) or E2F1 antibody (Cell Signaling Technology, #3742). Normal Rabbit IgGs were used as a negative control (involved in the ChIP kit #56383), and Histone H3 specific antibody (involved in the ChIP kit #56383) was used as a positive control. The cell lysates were incubated with protein A/G modified beads (involved in the ChIP kit #56383) overnight at 4°C in a rotator. Co-immunoprecipitated DNA was extracted and purified after ChIP as the template for later PCR reactions. The information of all PCR primers we used in the Chip assay was listed in Supplementary Data 1.

### Electrophoretic mobility shifts assay (EMSA)

the corresponding primer pairs listed in Supplementary Data 1. The genomic DNA was extracted from A549 cells and was used as the template for probe amplification using primer pairs PlrpF/PlrpR or FAM-F/PlrpR. The optimized coding sequence of E2F1 (NP_005216.1) was inserted into the plasmid pGEX-4T-1 at BamHI/XhoI sites to be expressed by fusion with GST in *Escherichia coli* BL21 (DE3, TIANGEN, China). The GST-E2F1 fusion protein was purified based on the protocol of GST 4FF Sefinose (TM) Resin Kit (Sangon Biotech, China). 20 ng FAM labeled probes were incubated with GST-E2F1 fusion protein in a 20-μl reaction mixture at 25 °C for 30 min. For competition assays, 0.4 μg of the unlabeled specific probe or the nonspecific probe sperm DNA was added to the binding reaction mixture. Then the samples were loaded on 4.5% (w/v) native polyacrylamide gels for electrophoresis. The fluorescently labeled DNA was detected by Amersham ImageQuant 800 (GE, America). The sequences of all probes and PCR primers we used in the EMSA assay were listed in Supplementary Data 1.

### Human tissues and Immunohistochemistry (IHC)

Tissues from breast cancer patients treated with CDK4/6i were obtained by biopsy before receiving CDK4/6 inhibitor palbociclib. Ethical approval was obtained from Zhejiang Cancer Hospital (Ethical number: IRB-2020-431) and informed consent was obtained from all patients. The detailed clinical information of all these patients was listed in raw data datasets. Patients with a PFS longer than 12 months after receiving palbociclib were defined as responders, and patients with a PFS shorter than 12 months were defined as non-responders. Tissues were embedded with paraffin and then were cut into sections with a thickness of 5 μm. After deparaffinization, hydration, blocking, antibodies (Anti-LRPPRC, Abcam, ab97505, 1:1000) incubating, and DAB staining, the staining picture of tissue sections were scanned by a Pathology workstation (Olympus, Japan). Expression score was obtained according to the products of the percentage of positive cells (0, 0%-10% positive; 1, 11%-50% positive; 2, 51%-80% positive; 3, 81%-100% positive) and LRPPRC staining intensity (0, negative staining; 1, weak staining; 2, medium staining; 3, strong staining). Samples with an LRPPRC expression score greater than 4 were defined as high expression, and with a score less than 4 were defined as low expression. The information of other antibodies used in IHC were listed below: Anti-Ki67, Proteintech, 27309-1-AP, 1:500; Anti-CDK6, Proteintech, 14052-1-AP, 1:150; Anti-CASPASE 3, Cell Signaling Technology, #9662, 1:100.

### Metabolic phenotypes

For the OCR assay, LUAD cells were suspended at a concentration of 4 × 10^5^ cells/mL. 100 μL of cells were added to Seahorse 24-well plates. After being adhered to the wall, the cells were treated with gradient concentrations of GAA in 500 μL complete medium for 48 h. Then the medium was discarded, and the cells were washed with 1 mL of pre-warmed Seahorse medium. Oligomycin was added to determine the oxidative leak, and carbonyl cyanide m-chlorophenyl (CCCP) was added to stimulate the mitochondrial electron transport to the maximum. Finally, rotenone and antimycin A were added to measure extra-mitochondrial respiration. For ECAR analysis, LUAD cells were seeded on Seahorse 24-well plates and treated with GAA for 48 h. After washing, cells were cultured in the medium (2 mM glutamine, pH 7.35) and monitored every 10 min following successive administration of 10 mM glucose and inhibitors (1 μM oligomycin and 50 mM 2-deoxyglucose).

### Proteomics

Proteins extracted from cells were alkylated by dithiothreitol and iodoacetamide, then digested by trypsin overnight at 37 °C. Peptides were analyzed by the nanoLC-Q-Exactive system and all the raw files were searched against the UniProt human protein sequence database in Maxquant (version 1.6.14.0). Perseus (version 1.6.14.0) was used to conduct a statistical analysis of the fold change of protein groups in each group. The pathway enrichment analysis of differential expressed proteins was carried out using KEGG Orthology Based Annotation System (KOBAS, Version 3.0) and Database for Annotation, Visualization and Integrated Discovery (DAVID, Version 6.8).

### Metabolomics profiling

A549 cells were seeded into 6-well cell culture dishes and treated with DMSO or GAA for 48 h. Cells were collected after washing three times with pre-cooled PBS. 500 uL pre-cooled extractant (80% methanol aqueous solution, including internal standard) was added and mixed via vortex for 2 min. Cells were lysed by freezing and thawing in liquid nitrogen three times, then metabolites were extracted by centrifuge for 20 min at 4 °C, 13800 × g, and supernatants were transferred to new tubes for LC-MS/MS.

### Genetic dependency data and RNA expression data

CRISPR dependency data was analyzed using the online database 21Q4 public Avana dataset (https://depmap.org/). The gene dependencies were estimated for each gene and cell line by the CERES algorithm.

### Animal models

4-6 week-old female nude mice (BALB/c-Nu) and sterilized food were purchased from Hangzhou Medical College. All mice were housed in pathogen-free facilities in the Zhejiang Cancer Hospital animal facilities. All mice were fed with filtered water and sterilized food (#2212) under standard conditions (72 degrees Fahrenheit, 65% humidity, 12 hours light/12 hours dark cycles). In the A549 cell-generated subcutaneous tumor models, A549 cells were injected into nude mice subcutaneously and randomly divided into different groups. GAA liposomes were dissolved in PBS to a final concentration of 10 mg/mL, and each mouse received a dose of 200 μL by tail vein injection every three days. ribociclib was dissolved in 20% cyclodextrin solution, and each mouse received a dose of 100 mg/kg by intragastric administration every day and 5 times a week. In the PDX subcutaneous models, tumor tissue was chopped uniformly and injected into nude mice subcutaneously using a trocar. The next grouping and treatment strategy was consistent with the A549 cells-generated subcutaneous tumor model. The mice were sacrificed depending on the tumor size and the level of animal discomfort. The xenograft tumors were fixed with 10% formalin and subjected to further IHC analysis. The maximal tumor size permitted by our ethics committee is 1500 mm^3^. The maximal tumor size in our animal experiments did not exceed the permitted maximal tumor size. For simulated survival analysis, the tumor volume threshold (above which was defined death) in A549 cell-generated subcutaneous tumors was 150 mm^3^, and the tumor volume threshold in PDX models was 500 mm^3^. Two models showed a completely different growth rate in vivo.

### Statistics & Reproducibility

All statistical results were reported as the mean ± SEM or mean ± SD of three or more biological replicates. Representative images for fluorescence staining, IHC staining, TEM, and Immunoblotting were shown and independently repeated at least two times with a similar tendency and the detail repeat time has been described in figure legents. Analyses and graphical presentations were performed using GraphPad Prism (version 8.0) and SPSS (version 17.0). No statistical method was used to predetermine sample size and no data were excluded from the analyses. *P* values < 0.05 were considered to be statistically significant (*NS*, not significantly). In the in vivo experiments, mice were age, and weight randomized appropriately. In vitro experiments, randomization was not performed. The investigators were blinded to allocation during the IHC experiment using breast cancer samples and outcome assessment. In other experiments, investigators were not blinded to allocation during experiments and outcome assessment.

### Reporting summary

Further information on research design is available in the Nature Portfolio Reporting Summary linked to this article.

## Supplementary information

Supplementary Information

Description of Additional Supplementary Files

Supplementary Data 1

Supplementary Data 2

Supplementary Data 3

Supplementary Data 4

Supplementary Data 5

Supplementary Data 6

Reporting Summary

## Data Availability

All data generated or analyzed during this study are included either in this article or in the supplementary information files. The RIP sequencing data have been uploaded to the Gene Expression Omnibus (GEO) repository, the accession number is GSE204923. The WES data of H1299 and H1299Re cells have been uploaded to the SRA repository, the accession number is PRJNA953351. The mass spectrometry proteomics data of H1299 and H1299Re cells have been deposited to the ProteomeXchange Consortium, the accession number is PXD042216. The raw metabolomics data of A549 before and after GAA treatment (Supplementary Data 6) have been deposited to the Metabolomics Workbench database, the accession number is ST002719. The analysis of TCGA dada was performed on the online analysis website TIMER (https://cistrome.shinyapps.io/timer/). The GEO datasets were downloaded from the NCBI database (https://www.ncbi.nlm.nih.gov/geo/query/acc.cgi?acc=GSE101929, https://www.ncbi.nlm.nih.gov/geo/query/acc.cgi?acc=GSE31852, and https://www.ncbi.nlm.nih.gov/geo/query/acc.cgi?acc=GSE19188). The analysis of public chip-seq data was performed in the online analysis website Cistrome (http://dbtoolkit.cistrome.org/). The analysis of the conserved nucleotide-binding motif of E2F1 protein at the *LRPPRC* promoter was performed in the online analysis website PROMO (https://alggen.lsi.upc.es/cgi-bin/promo_v3/promo/promoinit.cgi?dirDB=TF_8.3). Source data are provided with this paper.

## Source data

Source Data
