# Supplementary material for: The RNA-binding protein LRPPRC promotes resistance to CDK4/6 inhibition in lung cancer
Source: Nat Commun. 2023 Jul 14;14:4212. doi: 10.1038/s41467-023-39854-y (PMC10349134; doi:10.1038/s41467-023-39854-y)
Supplement: Supplementary file 1 — Supplementary Information [file 41467_2023_39854_MOESM1_ESM.pdf]

The RNA-binding protein LRPPRC promotes resistance to CDK4/6 inhibition in lung cancer

Wei Zhou,[1, 2, 6] Wenxi Wang,[1, 4, 6] Yuxin Liang,[2, 3, 6] Ruibing Jiang,[1] Fensheng Qiu,[1] Xiying Shao,[1] Yang Liu,[2, 3] Le Fang,[1, 4] Maowei Ni,[1] Chenhuan Yu,[1] Yue Zhao,[1] Weijia Huang,[1] Jiong Li,[5] Michael J Donovan,[1] Lina Wang,[2, 3] Juan Ni,[1] Dachi Wang,[1] Ting Fu,[1] Jianguo Feng,[1] Xiaojia Wang,[1] Weihong Tan,[1, 4, *] and Xiaohong Fang[1, 2, 3, 4, *]

1 Hangzhou Institute of Medicine (HIM), University of Chinese Academy of Sciences (Zhejiang Cancer Hospital), Chinese Academy of Sciences, Hangzhou, Zhejiang 310022, PR China.

2 Beijing National Research Center for Molecular Sciences, Institute of Chemistry, Key Laboratory of Molecular Nanostructure and Nanotechnology, Chinese Academy of Science, Beijing 100190, PR China.

3 University of Chinese Academy of Sciences, Beijing 100049, PR China.

4 School of Molecular Medicine, Hangzhou Institute for Advanced Study, UCAS, Hangzhou 310024, PR China.

5 Department of Medicinal Chemistry, Massey Cancer Center, Philips Institute for Oral Health Research, Virginia Commonwealth University, Richmond, Virginia, 23298-0540, United States.

6 These authors contribute equally.

*Correspondence: xfang@iccas.ac.cn, tan@hnu.edu.cn

Figure S1

a

Cell line	RB1 mutation	RB1 CNV
PC9	WT	Diploid
A549	WT	Diploid
H1299	WT	Diploid
H460	WT	Diploid
H1944	WT	Diploid
H23	WT	Diploid

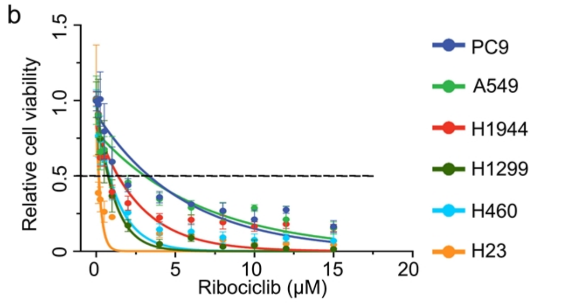

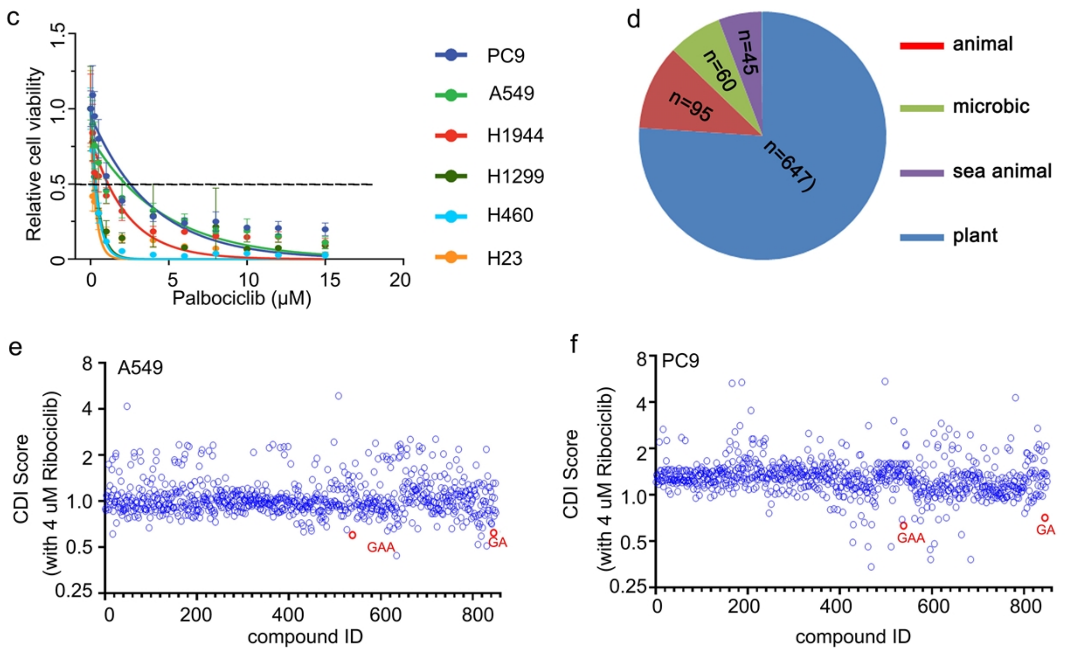

Fig.S1| Screening small molecules which increased the sensitivity against CDK4/6i in LUAD. a) Mutation information of *RB1* gene in different LUAD cell lines. All these cell lines we used had no mutation or copy number deletions in the *RB1* gene. **b, c**) Dose-response curves of LUAD cell lines to palbociclib or ribociclib (mean±SEM.; n=4 biological replicates). **d**) The information of the natural product library used in our screening work. Most of the active small molecules are extracted from plants. **e, f**) Screening results of A549 and PC9 cells with the natural compound library. GAA and GA were marked in red. Cells were treated with small molecule inhibitors alone or combined with CDK4/6i, then the synergy coefficient was calculated. A coefficient of drug interaction score (CDI) less than 0.8 was considered to have a synergistic effect. WT, wild type; CNV, copy number variation; GAA, gossypol acetate; GA, gossypol. Source data are provided as a Source Data file.

Figure S2

a

ID	CAS No	Formula	Structural formula	reported targets
Gossypol acetic acid	12542-36-8	$C_{32}H_{34}O_{10}$		Bcl-2/Bcl-xL/Mcl-1 APEX1 LRPRPC
Harmol	487-03-6	$C_{12}H_{10}N_2O$		NA
Farlutin	71-58-9	$C_{24}H_{34}O_4$		ER PR
Catharanthine sulfate	70674-90-7	$C_{21}H_{26}N_2O_6S$		BCL2 mAChR AChE nAChR
Rebaudioside A	58543-16-1	$C_{44}H_{70}O_{23}$		KATP channels
griffonilide	61371-55-9	$C_8H_8O_4$		NA
VERBENALIN	548-37-8	$C_{17}H_{24}O_{10}$		NA
Gossypol	303-45-7	$C_{30}H_{30}O_8$		Bcl-2/Bcl-xL/Mcl-1 APEX1 LRPRPC

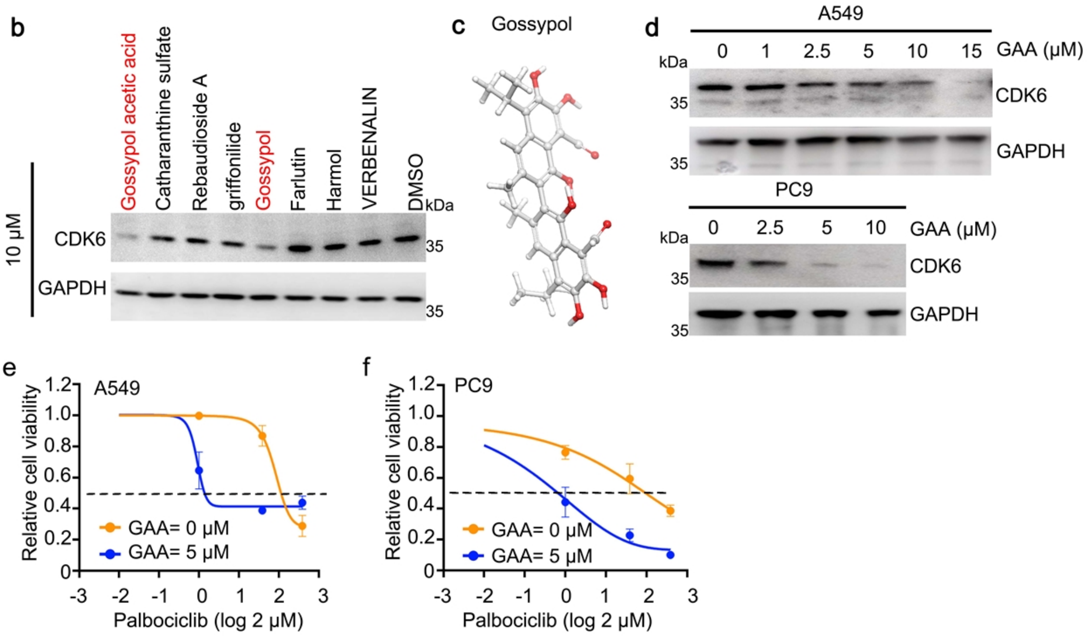

Fig.S2| GAA was screened out to decrease CDK6 expression and increase CDK4/6i sensitivity in primary resistance. a) Eight small molecules with synergistic effects with CDK4/6i were screened from 850 small molecules. The figure shows the name, structural formula, and reported protein targets of each small molecule. **b)** Immunoblotting of CDK6 in A549 cells. A549 cells were pretreated with indicated compounds in a concentration of 10 μM for 48 hours. **c)** Molecular structure of gossypol. Gossypol acetate is a medicinal form of gossypol with a non-covalently bound acetic acid. **d)** Immunoblotting of CDK6 in LUAD cell lines treated with GAA

in indicated concentrations for 48 hours. **e, f)** Dose-response curves of A549 and PC9 cells to CDK4/6i palbociclib with or without GAA treatment (mean±SEM.; n=4 biological replicates). The Immunoblotting results in figure b were a representative result of n = 3 independent experiments and image d was a representative result of n =2 independent experiments with the same tendency. Source data are provided as a Source Data file.

Figure S3

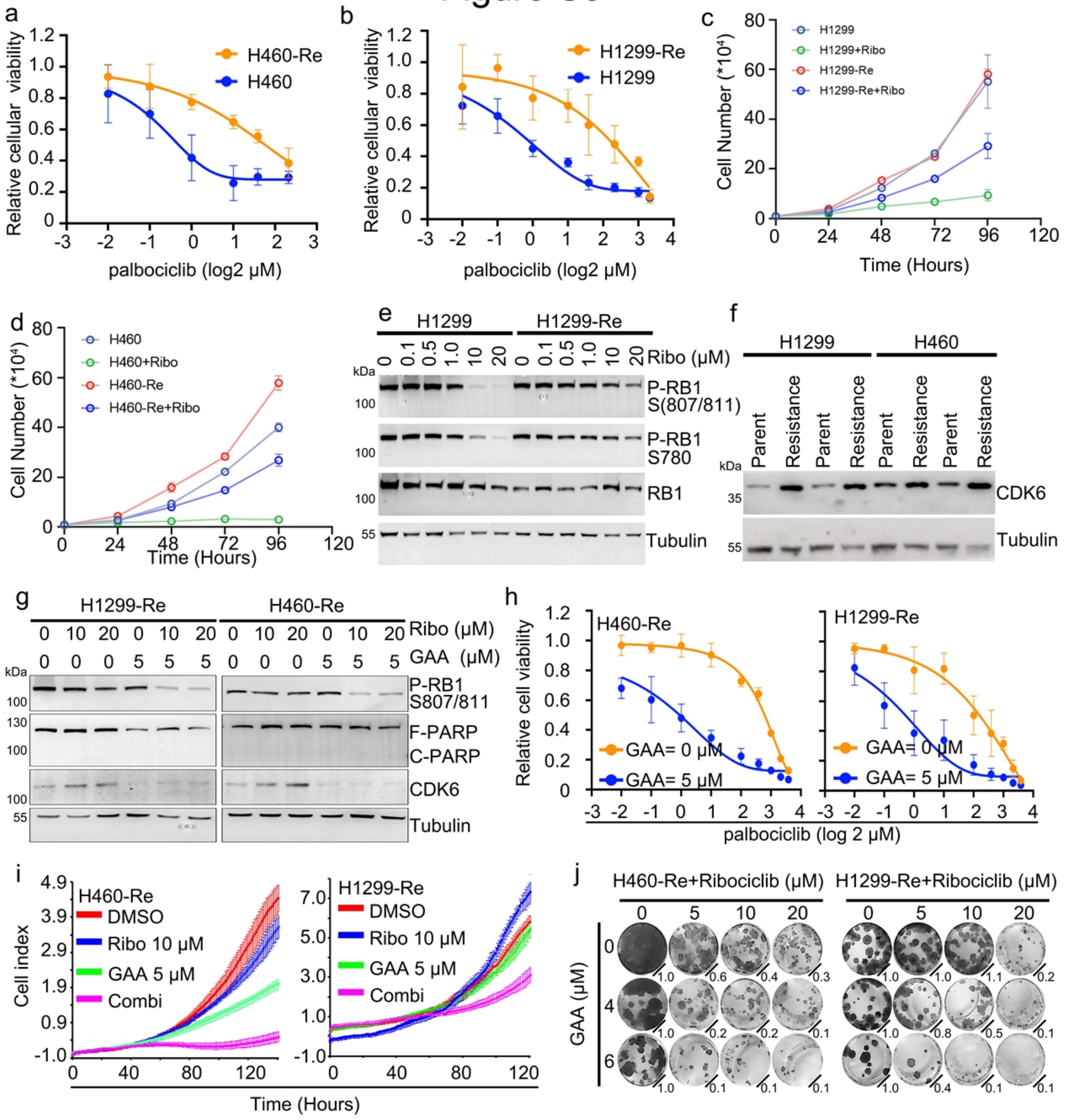

Fig.S3| GAA decreased CDK6 expression and restored CDK4/6i sensitivity in CDK4/6i acquired resistance models. a, b) Dose-response curves of parent cells (H460 and H1299) and acquired resistance cells (H460-Re and H1299-Re) to palbociclib (mean±SEM.; n=4 biological replicates). **c, d)** Cell proliferation curve of parent cells (H460 and H1299) and acquired resistance cells (H460-Re and H1299-Re) with or without ribociclib (Ribo, 10 μM). Cells were seeded in a 12-well plate at a density of 100,000 per well, and the cell numbers were counted every 24 hours. **e)** Immunoblotting of phosphorylated RB1 in H1299 and H1299-Re treated with ribociclib (Ribo) in indicated concentrations. **f)** Immunoblotting of CDK6 in parent cells and acquired resistance cells. **g)** Immunoblotting of indicated proteins in CDK4/6i acquired resistance models (H1299-Re and H460-Re) treated with indicated drugs. F-PARP, full-length PARP; C-PARP, cleaved PARP. **h)** Dose-response curves of CDK4/6i acquired resistance cells (H460-Re and H1299-Re) to palbociclib with or without GAA treatment (mean±SEM.; n=4 biological replicates). **i)** Cell proliferation

curves of CDK4/6i acquired resistance models (H460-Re and H1299-Re) treated with GAA, ribociclib (Ribo), or the combination (Combi). **j)** Representative clone pictures of CDK4/6i acquired resistance models (H1299-Re and H460-Re) treated with GAA, ribociclib or the combination. Normalized crystal violet staining intensity (mean; n=3 biological replicates) was also displayed. The results in figure e, f, j, and g were representative results of n = 3 independent experiments with similar results. Source data are provided as a Source Data file.

Figure S4

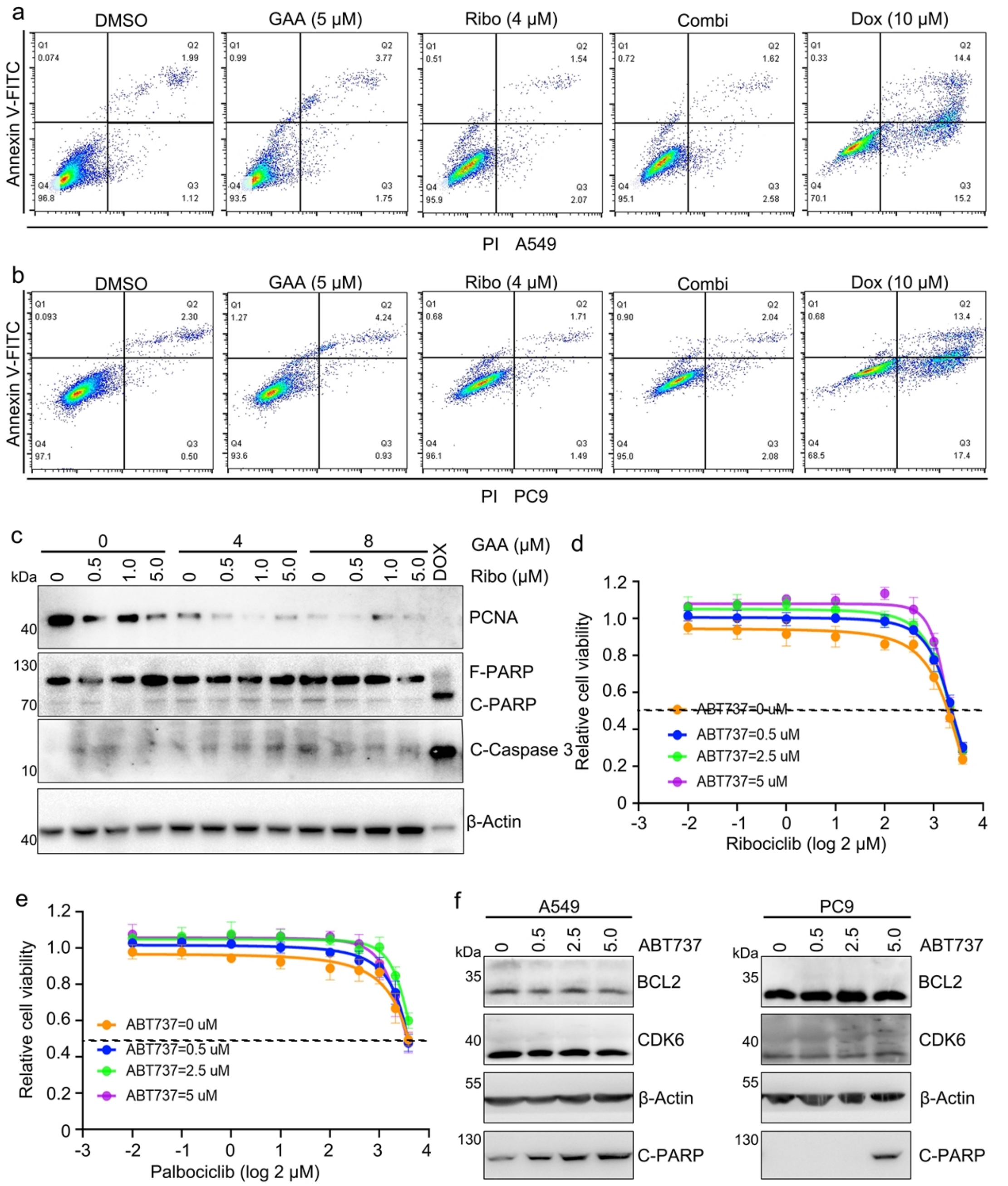

Fig.S4| GAA affected CDK6 expression and CDK4/6 inhibitor sensitivity independent of BCL2 protein and apoptosis. a, b) Representative Flow cytometry Annexin V and PI double-staining picture of A549 and PC9 cells treated with GAA, ribociclib (Ribo), or combination (combi). Dox treatment was used as the positive control for apoptosis. The Flow cytometry gating strategy was shown in supplemental Figure S10b. **c)** Immunoblotting of proliferation markers PCNA (Proliferating Cell Nuclear Antigen), apoptosis markers cleaved Caspase-3 (C-Caspase-3), full-length Poly(ADP-Ribose) (F-PARP), and cleaved PARP (C-PARP) in A549 cells treated with indicated drugs. **d, e)** Dose-response curves of A549 cells to ribociclib and palbociclib with or without indicated concentration of BCL2 inhibitor ABT737 (mean±SEM.; n=4 biological replicates). **f)** Immunoblotting of indicated proteins in

LUAD cell lines treated with different concentrations of ABT73. The results in figure a and b were representative results of n = 4 independent experiments with the same tendency. The Immunoblotting results in figure c, and f were representative results of n = 3 independent experiments with the same tendency. Source data are provided as a Source Data file.

Figure S5

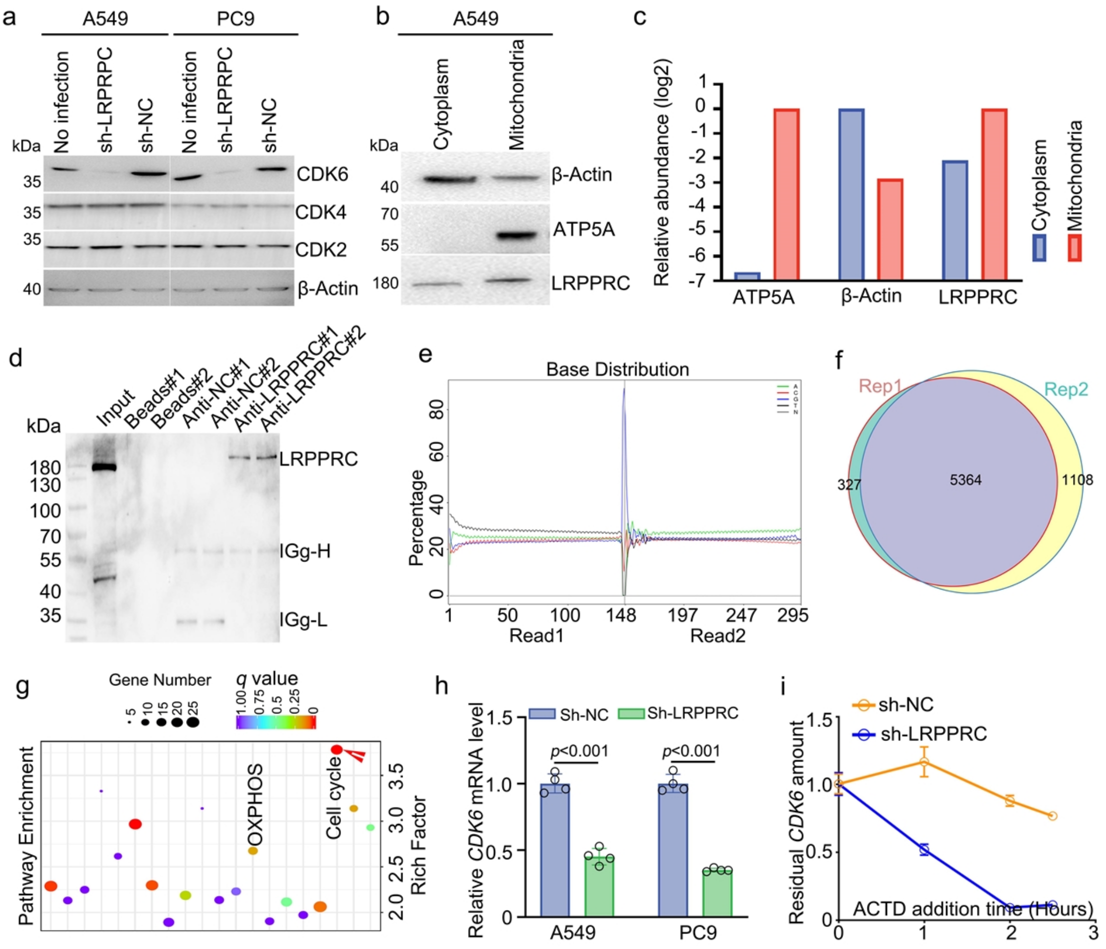

Fig.S5| LRPPRC regulated the expression of CDK6 protein by binding to *CDK6* mRNA. a) Immunoblotting of LRPPRC and indicated proteins in two LUAD cell lines before and after LRPPRC stable knockdown. **b, c)** Immunoblotting of indicated proteins in mitochondrial and cytoplasmic components of A549 cells. The gray value of each band was further quantified by Image J software. **d)** Immunoblotting of indicated protein in RIP produces. **e)** The percentage of different bases in RIP-seq clean data to the total bases. **f)** Overlap of LRPPRC-binding transcripts revealed from RIP-seq of two biological replicates. **g)** KEGG enrichment analysis results of LRPPRC binding genes. The cell cycle pathway showed the lowest *p*-value and the largest enrichment score. **h)** Real-time PCR quantification of the mRNA level of *CDK6* in LUAD cells before and after LRPPRC knockout (mean±SEM.; n=4 biological replicates). **i)** Representative quantified CDK6 mRNA degradation rate in A549 cells before and after LRPPRC knockdown. Actinomycin D (ACTD) was added to block RNA transcription, and RNAs were collected at different time points after ACTD addition. mRNA levels were represented as mRNA remaining after transcription inhibition (mean±SEM.; n=4 technical replicates). The Immunoblotting results in figure a were representative results of n = 3 independent experiments, and the results in figure b and d were representative results of n = 2 independent experiments with the same tendency. The PCR results in figure i were representative

results of n = 3 independent experiments with the same tendency. Statistical significance of quantification results in figure i was determined by a 2-way ANOVA analysis. The statistical significance of quantification results in figure h was determined by a two-tailed unpaired Student's *t*-test. Source data are provided as a Source Data file.

Figure S6

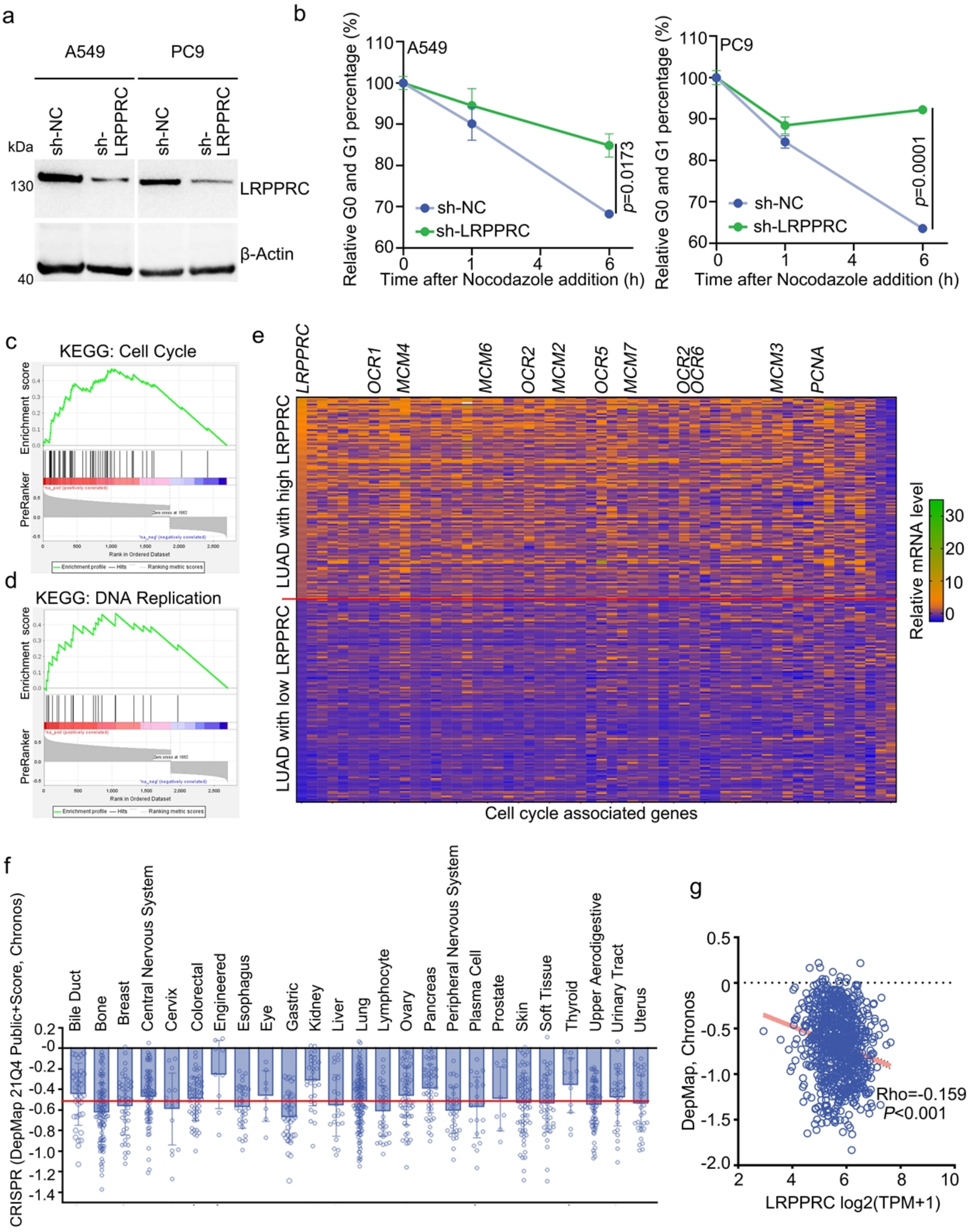

Fig.S6| LRPPRC promoted G1/S transition and cell proliferation. a) Immunoblotting of LRPPRC in two LUAD cell lines infected with control lentivirus (sh-NC) or lentivirus expressing LRPPRC specific shRNA (sh-LRPPRC). **b)** Normalized percentage of cells in G0 or G1 phase in LUAD cell lines before and after LRPPRC knockdown (mean±SEM.; n=3 biological replicates). Nocodazole was added to arrest cells at the G2/M phase, and cells were harvested at the indicated time point. The ratio of cells in the G1 or G1 phase at each time point was normalized to the value of 0 hours. **c, d)** GSEA results revealed a positive correlation between

LRPPRC expression and cell cycle and DNA replication processes in LUAD samples. The RNA expression data used for GSEA analysis were downloaded from the TCGA database. **e)** Heatmap of the mRNA expression level of *LRPPRC* and cell cycle-G1/S transition-associated genes in LUAD samples with high or low *LRPPRC* expression levels. **f)** Scatter plots of DEMETER score (DepMap CRISPR; Public 21Q4) of *LRPPRC* in different cell lines. In the figure, different cell lines were classified by tissue source (mean±SEM). The DEMETER score and *LRPPRC* expression data were downloaded from the public databases Depmap portal (mean±SEM, https://depmap.org/.). **g)** Correlation analysis of Chronos score and expression for *LRPPRC*. All expression values are in log2(TPM + 1). TPM, transcripts per million. The statistical significance of quantification results in figure b was determined by a 2-way ANOVA analysis by comparison at the endpoint. The Immunoblotting results in figure a were representative results of n = 2 independent experiments. The statistical significance of the correlation between the *LRPPRC* mRNA level and the Chronos score in figure g was determined by a two-tailed nonparametric Spearman correlation analysis. Source data are provided as a Source Data file.

Figure S7

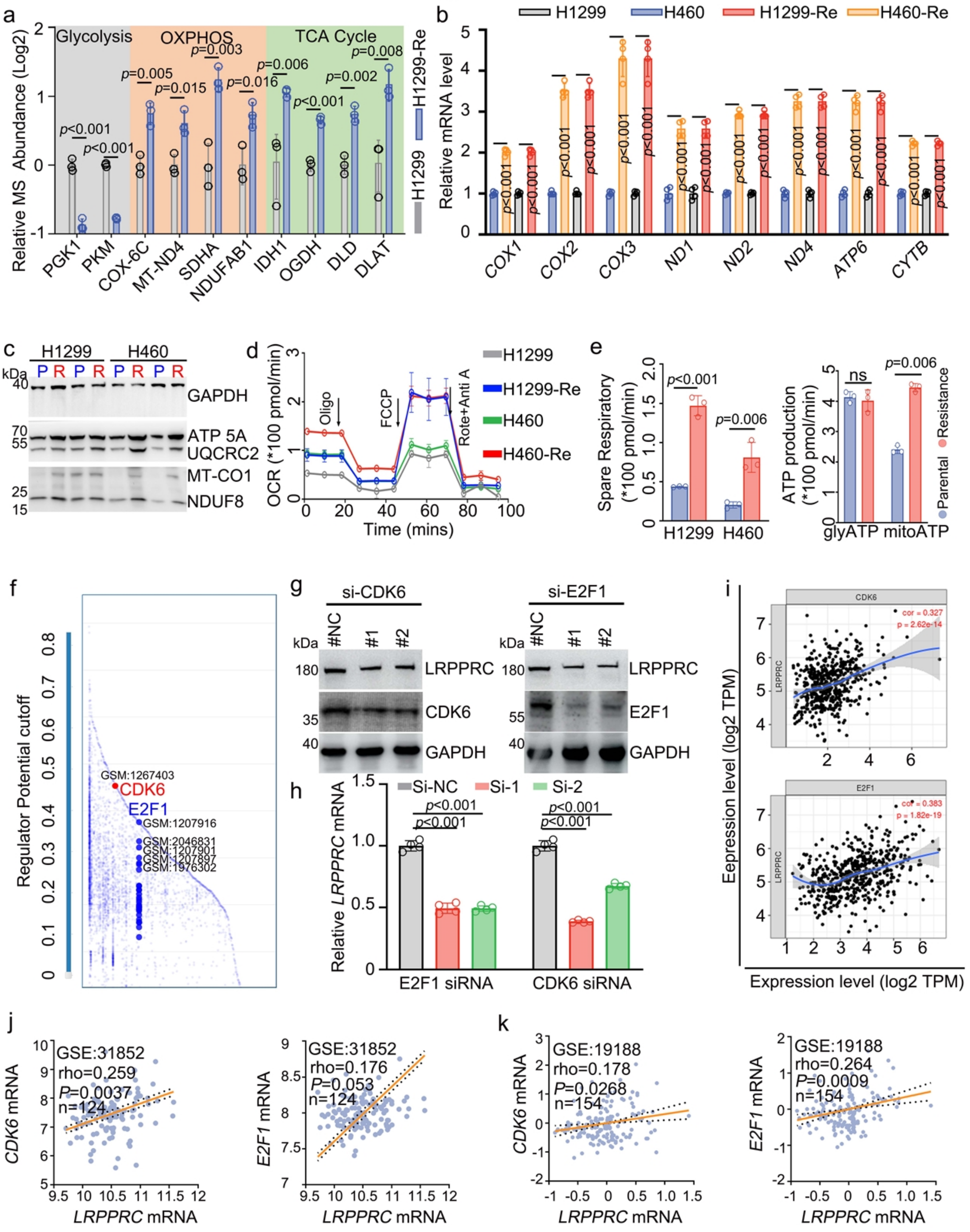

Fig.S7| CDK6 and E2F1 promote LRPPRC expression and enhance OXPHOS. a) Statistical results of the MS abundance of proteins involved in glycolysis, oxidative phosphorylation, and TCA cycle in H1299 and H1299-Re. Protein abundance was quantified by non-labeled MS, (mean±SEM.; n=3 biological replicates). **b)** Real-time PCR quantification of transcripts of OXPHOS complex subunits encoded by the mitochondrial genome in H1299, H460, and corresponding acquired resistance cells (mean±SEM.; n=4 biological replicates). **c)** Immunoblotting of OXPHOS complex subunits in parental cells (P) and acquired CDK4/6i-resistant cells (R). **d, e)** OCR

assay (Oxygen Consumption Rate) in parental cells and acquired CDK4/6i-resistant cells. Oligomycin was introduced at 20 minutes, FCCP was introduced at 48 minutes, and a mixture of rotenone and antimycin was introduced at 70 minutes. The Spare Respiratory and Mitochondria ATP yield ability (mitoATP) was further quantified (mean±SEM.; n=3 biological replicates). **f)** Statistics figure of regulatory potential of differential transcription factors against LRPPRC. The regulatory potential is a score to estimate how possible the factor can regulate LRPPRC. Y-axis represents the potential regulatory score, and the X-axis represents different factors. Dots in an X-axis line means the same factor. The dots of CDK6 and E2F1 were highlighted in red and blue, respectively. **g)** Immunoblotting of LRPPRC in PC9 cell line transfected with siRNA specific to CDK6 or E2F1. **h)** Real-time PCR quantification of *LRPPRC* mRNA in LUAD cell lines before and after knockdown of either CDK6 or E2F1 (mean±SEM.; n=4 biological replicates). **i)** Correlation analysis between the mRNA level of *LRPPRC* and *CDK6* or *E2F1* in LUAD samples in TCGA using the online website TIMER (https://cistrome.shinyapps.io/timer/). **j, k)** Correlation analysis between the mRNA level of *LRPPRC* and *CDK6* or *E2F1* in LUAD samples provided by two GEO datasets (GSE:31852, and GSE:19188). The Immunoblotting results in figure c and g were representative results of n = 3 independent experiments. The statistical significance of quantification results in figure h was determined by a 1-way ANOVA analysis. The statistical significance of quantification results in figure a, b, and e was determined by a two-tailed unpaired Student's *t*-test. The correlation analysis in figure i, j, and k was determined by a two-tailed nonparametric Spearman correlation analysis. Source data are provided as a Source Data file.

Figure S8

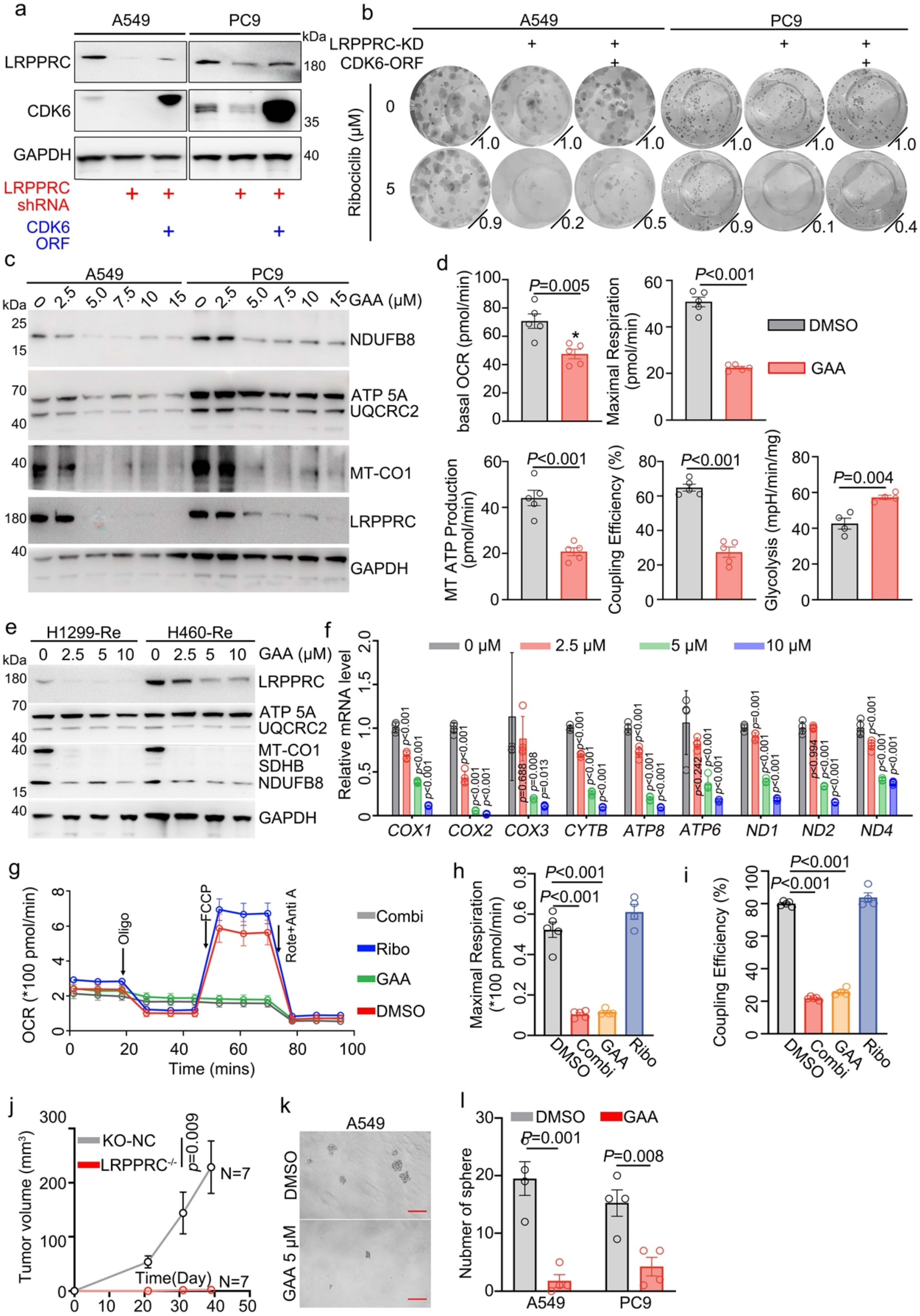

Fig.S8| GAA also suppresses OXPHOS and eliminates cancer stem cells. a) Immunoblotting of CDK6 and LRPPRC in LUAD cell lines transfected with LRPPRC specific shRNA and CDK6 overexpression plasmid (CDK6 ORF). **b)** Representative clone pictures of A549 and PC9 cells transfected with

LRPPRC-specific shRNA and CDK6 overexpression plasmid and then treated with ribociclib. Relative colony number in each group was also shown (mean; n=3 biological replicates). **c)** Immunoblotting of OXPHOS complex subunits in A549 and PC9 cells treated with GAA in different concentrations. **d)** Quantification of basal respiration, Mitochondria ATP (MT ATP) production, maximal respiration, coupling efficiency, and glycolysis for different A549 cells pretreated with 10 μM GAA or DMSO control (mean±SEM.; n=5 biological replicates in the analysis of basal respiration, MT ATP production, maximal respiration, and coupling efficiency; n=4 biological replicates in the analysis of glycolysis). **e)** Immunoblotting of OXPHOS complex subunits in H1299-Re and H460-Re cells treated with different GGA concentrations. **f)** Real-time PCR quantification of transcripts of OXPHOS complex subunits encoded by the mitochondrial genome in H1299-Re cells treated with different concentration GAA (mean±SEM.; n=4 biological replicates). **g-i)** OCR detection in H1299-Re cell treated with GAA (5 μM), ribociclib (Ribo, 10 μM), or combination (Combi). The maximal respiration and coupling efficiency were further quantified (mean±SEM.; n=4 biological replicates in GAA and ribociclib treatment group, n=5 biological replicates in DMSO and combination treatment group.). **j)** Tumor growth curves of xenografts using control A549 and LRPPRC knockout A549cells (mean±SEM.; n=1 experiment; n=7 mice in each group). **k, l)** Representative images of tumorspheres generated from A549 cells treated with GAA or DMSO control. The tumorspheres formation ability was quantified and presented as the number of tumorspheres per 1000 cells (mean±SEM.; n=3 biological replicates). The results in figure a, b, c, and e were representative results of n = 3 independent experiments with the same tendency. The statistical significance of quantification results in figure f, h, and i was determined by a 1-way ANOVA analysis. The statistical significance of quantification results in figure d and l was determined by a two-tailed Student's *t*-test. The statistical significance of quantification results in figure j was determined by a 2-way ANOVA analysis at the endpoint. Source data are provided as a Source Data file.

Figure S9

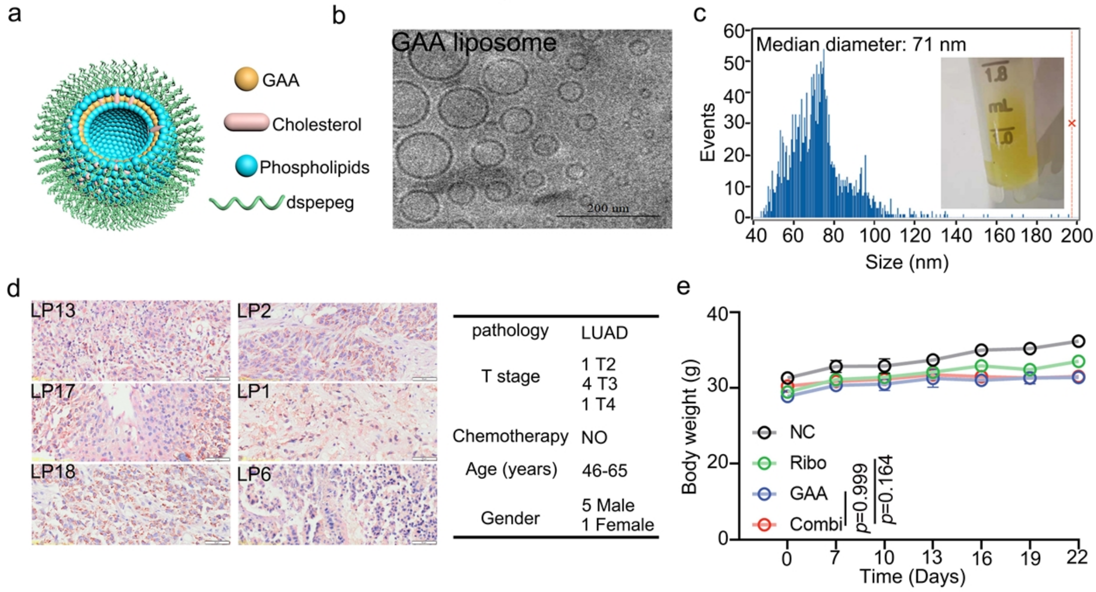

Fig.S9| GAA inhibited the LRPPRC-CDK6 feedback loop and increased CDK4/6i sensitivity *in vivo*. a) Schematic diagram of liposome encapsulating GAA. **b)** TEM pictures of GAA-loaded liposomes. **c)** Nano flow cytometry analysis of GAA nanoliposomes. **d)** Representative IHC pictures of LRPPRC protein in 6 lung adenocarcinoma PDX models and the summary of clinical information of the 6 lung adenocarcinoma PDX models. **e)** Bodyweight change curve of PDX animal models treated with indicated drugs (mean±SEM.; n=1 experiment; n=7 mice in the control group (NC), n=6 mice in GAA treatment group (GAA), n=5 mice in ribociclib treatment group (Ribo), and n=6 mice in the combination treatment group (Combi)). The TEM results in figure b were representative results of n = 2 independent experiments with the same tendency. The IHC results in figure d were representative results of n = 3 independent experiments with the same tendency. The statistical significance of quantification results in figure e was determined by a 2-way ANOVA analysis by comparison of the body weight data at the endpoint. Source data are provided as a Source Data file.

Figure S10

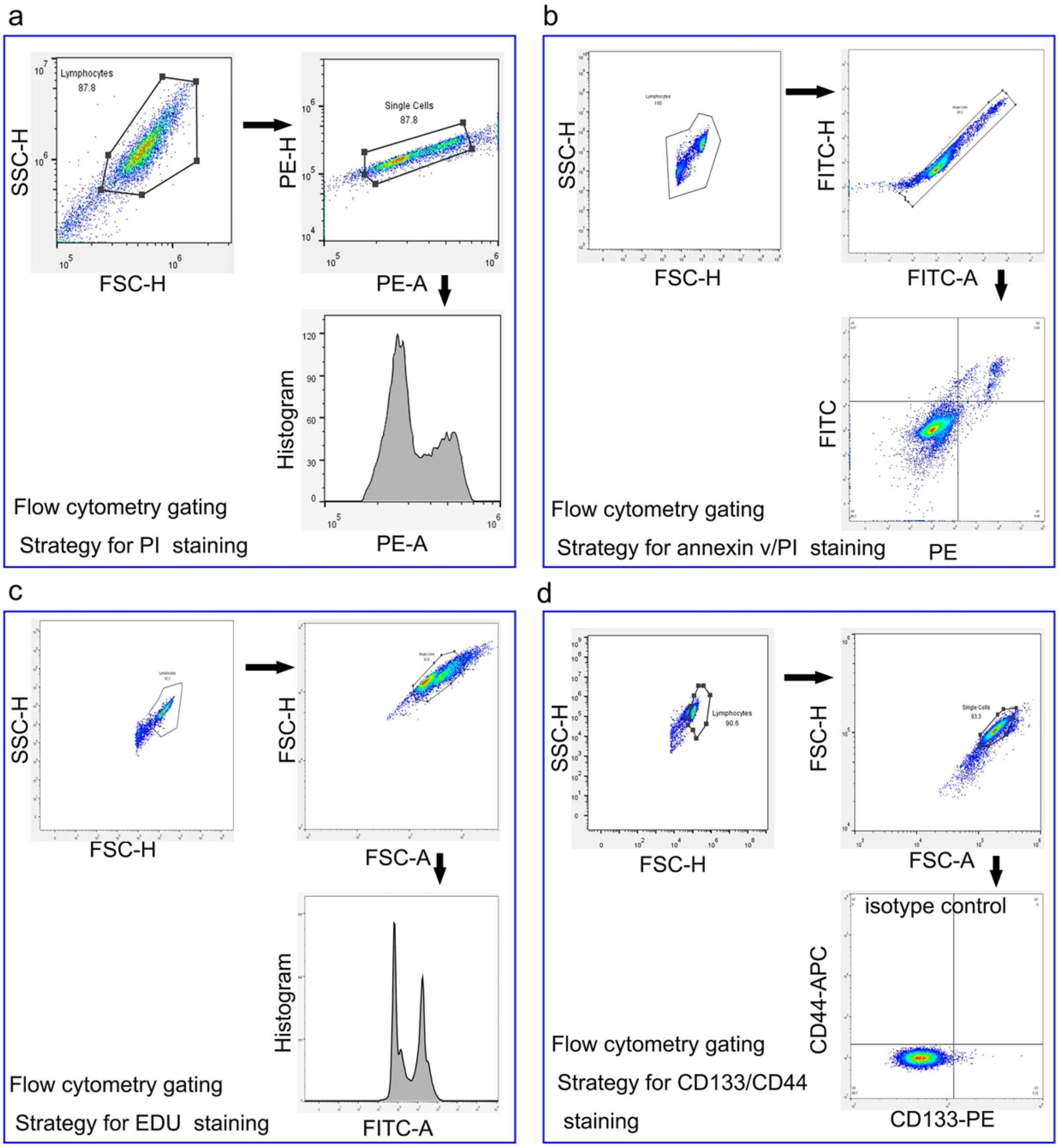

Fig.S10| Gating strategy for flow cytometry. a) Gating strategy for PI staining. The major cell population was first delineated by FSC and SSC. Then, the single-cell population was delineated by PE-A and PE-H. Finally, the fluorescence signal of the single-cell population was analyzed. **b)** Gating strategy for Annexin v/PI staining. All cells detected by FSC and SSC were subjected to further analysis. Then, the single-cell population was delineated by FITC-A and FITC-H. Finally, the fluorescence signal of the single-cell population was analyzed. **c)** Gating strategy for EDU staining. The major cell population was first delineated by FSC and SSC. Then, the single-cell population was delineated by FSC-H and FSC-A. Finally, the fluorescence signal of the single-cell population was analyzed. **d)** Gating strategy for CD133 and CD44 staining. The major cell population was first delineated by FSC and SSC. Then, the single-cell population was delineated by FSC-H and FSC-A. Finally, the fluorescence signal of the single-cell population was analyzed.
