# Supplementary material for: The RNA-binding protein LRPPRC promotes resistance to CDK4/6 inhibition in lung cancer
Source: Nat Commun. 2023 Jul 14;14:4212. doi: 10.1038/s41467-023-39854-y (PMC10349134; doi:10.1038/s41467-023-39854-y)
Supplement: Supplementary file 2 — Description of Additional Supplementary Files [file 41467_2023_39854_MOESM2_ESM.doc]

File Name: Supplementary Data 1

Description: Sequences of oligonucleotides and primers used in this work.

File Name: Supplementary Data 2

Description: List of LRPPRC-binding transcripts (FPKM>30).

File Name: Supplementary Data 3

Description: Differentially expressed protein in H1299 and H1299-Re cell lines detected by

Proteomics.

File Name: Supplementary Data 4

Description: KEGG Pathway enrichment of differentially expressed proteins (H1299-Re/H1299).

File Name: Supplementary Data 5

Description: Quantification of the abundance of intermediate metabolite of A549 cells treated with GAA or DMSO.

File Name: Supplementary Data 6

Description: The raw metabolomics data of A549 before and after GAA treatment.
